# Alginate-Based Materials Loaded with Nanoparticles in Wound Healing

**DOI:** 10.3390/pharmaceutics15041142

**Published:** 2023-04-04

**Authors:** Anna Froelich, Emilia Jakubowska, Monika Wojtyłko, Barbara Jadach, Michał Gackowski, Piotr Gadziński, Olga Napierała, Yulia Ravliv, Tomasz Osmałek

**Affiliations:** 1 Chair and Department of Pharmaceutical Technology, Poznan University of Medical Sciences, 6 Grunwaldzka Street, 60-780 Poznań, Poland; 2 Department of Pharmacy Management, Economics and Technology, I. Horbachevsky Ternopil National Medical University, 36 Ruska Street, 46000 Ternopil, Ukraine

**Keywords:** wound, alginate, nanoparticles, healing, wound dressings

## Abstract

Alginate is a naturally derived polysaccharide widely applied in drug delivery, as well as regenerative medicine, tissue engineering and wound care. Due to its excellent biocompatibility, low toxicity, and the ability to absorb a high amount of exudate, it is widely used in modern wound dressings. Numerous studies indicate that alginate applied in wound care can be enhanced with the incorporation of nanoparticles, revealing additional properties beneficial in the healing process. Among the most extensively explored materials, composite dressings with alginate loaded with antimicrobial inorganic nanoparticles can be mentioned. However, other types of nanoparticles with antibiotics, growth factors, and other active ingredients are also investigated. This review article focuses on the most recent findings regarding novel alginate-based materials loaded with nanoparticles and their applicability as wound dressings, with special attention paid to the materials of potential use in the treatment of chronic wounds.

## 1. Introduction

Wounds are considered as an injury of external body barriers, e.g., skin or mucous membranes, as well as deeper tissues, upon the impact of external factors, including heat, aggressive chemicals, or mechanical damage. Depending on the clinical characteristics, wounds can be classified as acute or chronic, and each type demands particular management. Acute wounds can occur as a result of surgical intervention or an injury, while chronic ones are usually associated with comorbidities, including diabetes, vascular disease and infections. An important type of chronic wound is pressure ulcer, occurring in elderly patients with physical impairments and confined to bed or chair, which results in prolonged pressure [1]. In elderly hospitalized patients, it is associated with 25–33% higher mortality rate [2]. It is noteworthy that chronic wounds in particular significantly affect the quality of life, being a cause of physical pain and numerous psychological difficulties related to distress, depression, anxiety, and social withdrawal [3,4,5]. Moreover, without proper management, they can increase in size and lead to severe bacterial infections, being also a cause of amputation [6], sepsis development or premature death [7]. On the other hand, wound management is an important socioeconomic issue, especially when chronic conditions are taken into consideration. It is estimated that in the United States, open wounds affect about 3% of patients over 65 years of age, and approximately 2% of the total population suffer from chronic wounds [6,8]. The data estimated with the use of the Medicare system [9] indicate that the costs related to wound management are enormous and, in 2014, could reach approximately USD 100 billion. The largest part of the costs related to wound treatment seems to be associated with hospitalization [10], which is necessary whenever serious complications occur and a surgical intervention is needed. An appropriate therapeutic approach in wound management is crucial to avoid bacterial infection and the development of further problems, which is particularly challenging in patients suffering from diabetes and obesity, as well as in elderly population more frequently revealing poor nutritional status or impaired immunological response. 

Depending on the type of wound, overall patient health and occurring comorbidities, an appropriate therapeutic strategy should be selected to provide the conditions needed for a fast and efficient healing process. For chronic wounds, the TIME approach for the evaluation of a wound condition is applied. In the first step, the tissue (T) assessment and debridement must be performed; next, infection and inflammation (I) are evaluated, followed by moisture (M) checking and management of wound exudate. Finally, the edge (E) of the wound is checked [11]. However, wound healing requires a complex approach involving a careful consideration of other health factors, including the management of comorbidities influencing the process or the general nutritional status of the patient. One of the important elements of proper wound care is wound dressing selection, which should be performed based on the wound type, depth, location, and the presence or absence of exudate and infection symptoms. In order to provide a good environment for wound healing, the dressing material should display a few important features, with the ability to maintain a moist environment as one of the necessary ones. Moreover, good dressing materials should also absorb the excessive amount of exudate, allow gas transmission, and provide the protection from external forces that could damage newly formed tissue, as well as from microorganisms that could colonize the wound. All materials contacting the wound should be biocompatible and nontoxic. Finally, other properties, such as cost-effectiveness and easy handling, should be taken into consideration [12].

**Figure 1:**
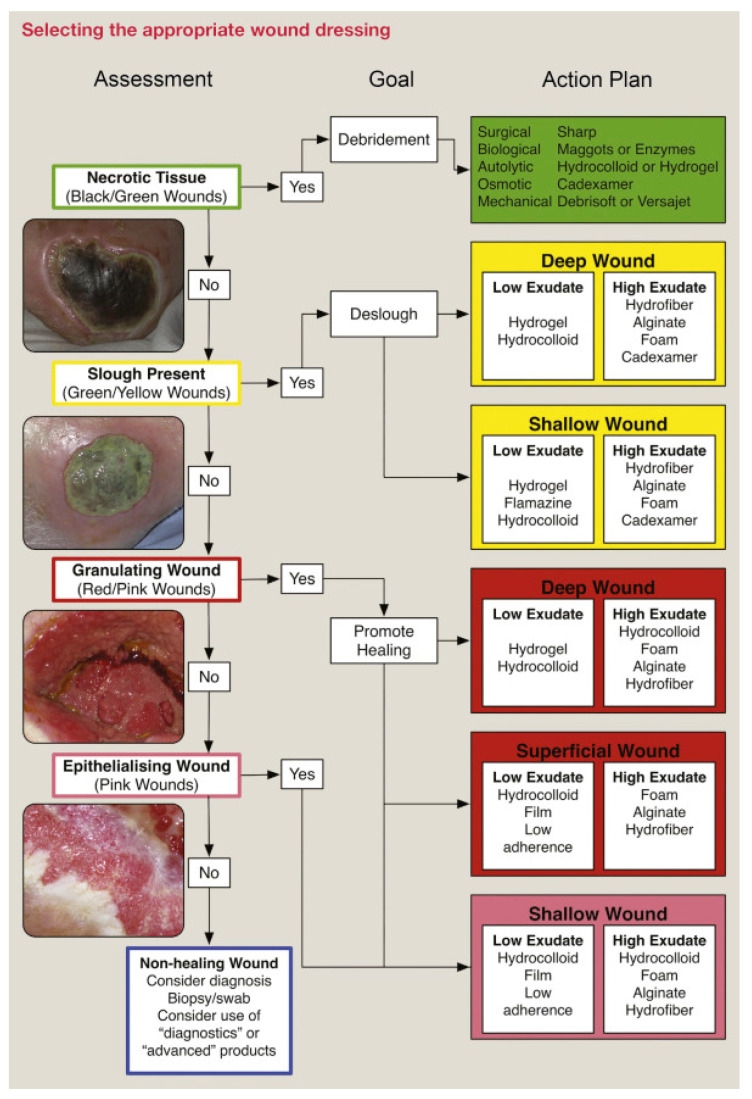
Selection of the appropriate wound dressing. Reproduced from [13] with permission.

It is noteworthy that the application of traditional cotton-based dressings usually led to excessive drying and the adherence of the dressing material to the wound. Currently, a large number of alternatives to traditionally applied materials are available. It must be also emphasized that apart from the products successfully marketed and widely applied in clinical practice, the development of novel materials with improved properties is a subject of extensive scientific investigations. Modern wound dressings can have a form of a film, foam, or gel, depending on the depth of the wound, the amount of exudate, and the features of the wound bed. In the case of non-complicated surgical wounds, usually the dressing requirements are different from chronic or non-healing wounds [13]. The dressings widely applied in a contemporary medicine can be obtained from different materials, depending on the desired properties and potential applications. The classification of wound dressings based on the applied material source is presented in Figure 1. Taking into consideration the fact that the proper moisture level is crucial for wound healing, most of the modern dressings contain hydrogel-forming materials as basic components. This approach allows for absorbing the excessive amounts of discharge without drying the wound bed. 

Alginate is a polysaccharide widely recognized as safe, biocompatible, and biodegradable material applied in numerous areas, including food manufacturing [14], drug delivery systems [15], cosmetic industry [16], tissue engineering [17] and the manufacturing of the materials useful in wound management. It is noteworthy that the idea of alginate application in wound dressings is not new. Alginate-based dressings have been successfully marketed and utilized in wound management since the 1980s, when Sorbsan was introduced as a treatment in diabetic and trophic ulcers [18,19]. Since then, numerous different dressing brands have been manufactured and commercialized. The available products are manufactured in different shapes and sizes, in order to fit any individual needs. Alginate dressings can be used to treat superficial wounds and lacerations but also to feel the cavities frequently occurring in the development of pressure ulcers. It is important to note that as strongly absorbent material, alginate is recommended in the management of wounds with moderate to high amount of the exudate. Currently available products can also differ in terms of composition and properties, as combinations with other polymers or silver are applied. A few examples of the commercially available alginate-based products are presented in Table 1.

Alginate materials in wound management are also a subject of extensive research. As the polymer reveals many advantageous properties in terms biomedical applications, including also excellent biocompatibility and nontoxic character, its potential is still receiving a lot of interest and novel composite materials are investigated and developed. One of the most interesting and intensively explored options is the enrichment of alginate dressings with nanoparticles applied as carriers to therapeutic agents. In this way, the properties of the original dressing can be significantly modified and novel materials displaying interesting therapeutic features can be obtained, depending on the type of nanoparticles applied. So far, several silver-enhanced dressings with antibacterial properties have been introduced to the pharmaceutical market, including also alginate-based materials, as shown in Table 1. However, most of the commercially available products contain silver in an ionic form and there are only a few dressings with nanocrystalline silver, such as collagen-based Acticoat^®^ (Smith & Nephew; London, UK) [28]. Wound healing materials with incorporated nanoparticles offer some important advantages, including the possibility to extend the drug release process which, in turn, may result in reduced dressing change frequency. Moreover, in the case of active agents susceptible to degradation, the entrapment in nanoparticles may improve the stability. On the other hand, the development of nanomedicines is still challenging and concerns related to nanoparticle safety and accumulation in the body are frequently mentioned [29,30,31]. 

The aim of this review is to summarize and present the most important research directions, focusing on the design and characterization of novel alginate-based materials enriched with therapeutic nanoparticles. As it was already mentioned, wound management is an important healthcare issue, considering the fact that the number of patients suffering from chronic conditions associated with impaired wound healing is increasing worldwide. The development of novel, safe, and effective therapeutic strategies, as well as innovative materials supporting wound management, especially in patients with existing comorbidities, is an important challenge of contemporary medicine. Among numerous innovatory approaches towards more efficient wound management, dressings with embedded therapeutic nanoparticles seem to be the most promising one, as demonstrated by the commercial success of dressing materials with nanocrystalline silver. In this article, the most extensively explored research directions involving alginate dressings with solid nanoparticles are presented and discussed. 

## 2. Wounds: Pathophysiology, Classification, Therapeutic Approaches

According to the definition, a wound is an anatomical interruption of the continuity of external layers (skin, mucous membrane) and deeper tissues under the influence of a damaging physical, chemical, or biological factor [32]. The classic characteristics of a wound include pain, bleeding, and dripping [33]. Opening of the wound edges depends on its size, elasticity, and the direction of their separation [34]. In general, two types of wound are distinguished, superficial, whose depth does not exceed the subcutaneous tissue, and deep wounds, which go beyond. Further differentiation depends on the way the force works, the mechanism of its action [35]. Abrasions and scratches are usually caused by not very strong pressure of a hard, blunt tool, falling, or hitting a hard and rough surface. These wounds are shallow; only the epidermis and superficial layers of the skin are damaged [36]. Puncture wounds are caused by a long, narrow, and sharp object (e.g., nail, needle, pitchfork, bayonet) and can drill into body cavities, causing the damage of internal organs. These wounds do not gape, and their edges heal quickly. Depending on the depth, they can lead to internal bleeding [37]. Cut wounds are caused by sharp objects (e.g., a knife or glass). The edges and walls are even, and they usually bleed profusely [38]. Bruised wounds arise as a result of breaking the skin and deeper tissues as a result of the action of a blunt object, e.g., as a result of being hit with a hammer or a stone. The edges of the wound are then uneven, crushed, and swollen; there are bruises around the wound. Bleeding is much less than in cut wounds due to damage to the blood vessels. Broken tissues die quickly and then necrosis occurs [39,40]. Laceration arises as a result of violent contact with a curved tool, e.g., a hook. Its edges are uneven and jagged, and at the bottom, you can see jagged fat and muscle. Skin and deeper tissue loss is common [41]. Bite wounds are wounds inflicted by the teeth of domestic or wild animals. They have the character of bruised or lacerated wounds. Tissue damage is greater than just perforating the skin. These wounds heal poorly and are easily infected. If bitten by an animal, there is a risk of infection with the bacterial flora in the animal’s mouth. These wounds also carry the risk of rabies infection [42,43]. 

Healing is the biological process that takes place in a wound to close it and form a scar. The time and course of the wound healing process depend on many factors. The characteristics of the wound, i.e., its size, shape, type, amount of damaged tissues, foreign bodies, and secretion outflow conditions, have a large impact on the course of the wound healing process. An extremely important factor is also the blood supply to the wound area [44,45,46].

There are two main ways of wound healing. The most beneficial, immediate (primary, temporary) healing is when the edges of the wound are brought together and fuse, and a linear scar is formed. The delayed or secondary healing is called granulation, a longer process that occurs when the primary closure of the wound was not completed for various reasons. In such cases, granulation tissue is formed at the bottom of the wound, which is the basis for the regeneration of the superficial layers of the skin and epidermis. Such healing requires proper care and regular dressing changes. The scar resulting from healing by granulation is thicker and more visible [47,48,49].

Analyzing in more detail the course of wound healing, two successive and partially overlapping stages can be distinguished. The first is referred to as cleansing phase, during which the wound edges are swollen, red, and warm. The area is painful, and the wound is initially filled with clotted blood and liquid secretion. Over time, dead tissue separates from healthy one and is excreted from the wound. During the next stage, called recovery phase, swelling and redness gradually disappear and soreness decreases. The walls and bottom of the wound are gradually covered with granulation tissue, which over time fills the entire wound. The deeper layers of granulation tissue shrink, and the wound surface gradually decreases. The granulation tissue gradually becomes fibrotic, hardens, and shrinks many times and transforms into a scar [44,48]. A necessary condition for the proper healing of damaged skin is the appropriate course of each of the following stages: hemostasis, inflammation, proliferation, and remodeling [50]. As described in more detail, the following steps can be listed in the complete recovery process: rapid hemostasis → appropriate inflammation → mesenchymal cell differentiation, proliferation, and migration → suitable angiogenesis → prompt re-epithelialization → synthesis, cross-linking and alignment of collagen [51,52,53]. 

In the case of acute wounds, first, it is recommended to wash with water, and then disinfect it with an appropriate germicide, without iodine or alcohol content. The wound should be cleaned gently to avoid further tissue damage [54]. When the bleeding has stopped, the wound should be covered with sterile gauze or a special dressing. The dressing should be changed regularly, but not too often, as this may impede healing [13]. A moisturizing agent that supports healing and protects against infection can be used under the dressing. Proper and regular wound cleansing is the first most important rule in the treatment of acute wounds. The purpose is primarily to remove factors that may adversely affect the healing process or increase the risk of infection, and should be carried out gently and as accurately as possible. In order to clean the wound, the patient can use enzyme ointments, active gels, or rinsing and cleansing dressings [55,56]. Another important aspect is decontamination and infection prevention. Direct use of such agents as hydrogen peroxide [57], ethacridine lactate [58], ethanol [59], or gentian violet is not recommended, as it can lead to serious irritation. It is worth choosing an agent that is safe, stimulates healing, and does not destroy the forming granulation tissue and, at the same time, is effective, killing bacteria, viruses, and fungi [60]. Both at the beginning and throughout the healing process, the wound should be properly moisturized, as it enables migration of fibroblasts and proper reconstruction of the wound. It is stated that a wound in a moist environment heals up to twice as fast, and the risk of scarring is reduced [45,61,62]. 

It has to be taken into account that any wound, even superficial, can become chronic if it is not properly treated. In addition, malfunctions of the body, such as diabetes or obesity, have an impact on the wound healing process [63,64]. Therefore, in the case of any damage to the skin, it is important not to underestimate it and take care of proper hygiene. Otherwise, infection can easily set in. Moreover, even the best dressing and therapeutic agents will not ensure effectiveness if the basic conditions that ensure the body’s ability to regenerate are not met. Only proper treatment and ongoing monitoring of the wound condition can shorten the healing time. Leaving skin damage unattended or failing to provide appropriate conditions for healing means that any wound can become chronic [65]. The healing of such wounds is a complicated and time-consuming process, often requiring hospitalization or constant nursing care [66]. 

Skin recovery may be disturbed by a number of pathological factors, which are generally classified as local and systemic [67,68]. The first group includes: oxygen level [69], infection [70,71], or foreign body. The next includes factors such as age [72], gender [73], sex hormones [74], stress [75,76], ischemia [49], diseases (diabetes, keloids, fibrosis, hereditary healing disorders, jaundice, uremia) [77], obesity [78], pharmacotherapy (steroids, nonsteroidal anti-inflammatory drugs, chemotherapeutic agents) [79,80], alcohol abuse [81], smoking [82], and conditions with reduced immunity (cancer, radiation therapy, AIDS), and nutrition habits [83,84].

The most common types of chronic wounds include: chronic post-traumatic, radiation and cancer wounds, diabetic ulcers (diabetic foot), leg ulcers, bedsores, and burns. Especially, bedsores, diabetic foot syndrome, and venous leg ulcers are very embarrassing and painful and sometimes exclude the patient from society. 

In conclusion, it is necessary to refer to the general scheme of wound treatment, referred to by the acronym TIME, the individual stages of which include: T—tissue debridement (cleansing), which assumes careful inspection of the wound with subsequent removal of necrotic tissue along with all debris; I—infection and inflammation control, which includes the use of topical antiseptics with antimicrobial, antiviral, and anti-inflammatory effects; M—moisture balance; and E—edges, based on observation of the wound edges and stimulation of epidermal formation [85,86,87]. 

## 3. Alginate: Structure, Properties, and Biomedical Applications

Alginate is a polysaccharide occurring naturally in cell walls of brown marine algae and also synthesized by bacteria belonging to *Pseudomonas* and *Azotobacter* genera. The term is usually used in the descriptions referring to alginic acid and its salts [17]. 

**Figure 2:**
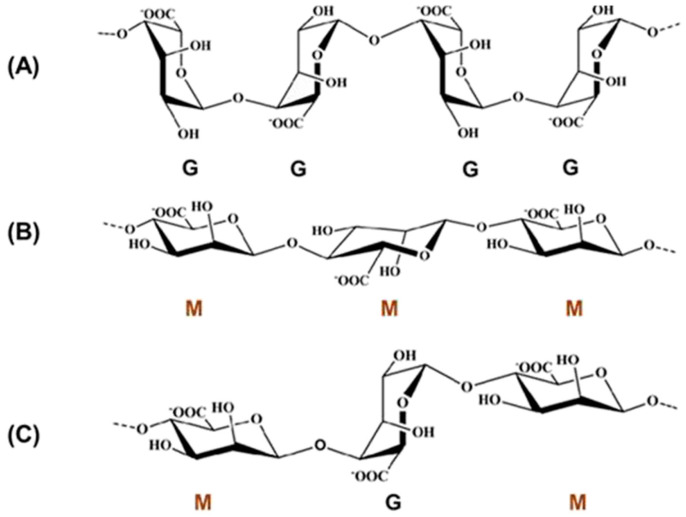
(**A**) Homopolymeric blocks of poly-α-1,4-l-guluronic acid (GG), (**B**) homopolymeric blocks of poly-β-1,4-d-mannuronic acid (MM), (**C**) heteropolymeric blocks of alternating M and G residues. Reproduced from [92] with permission.

**Figure 3:**
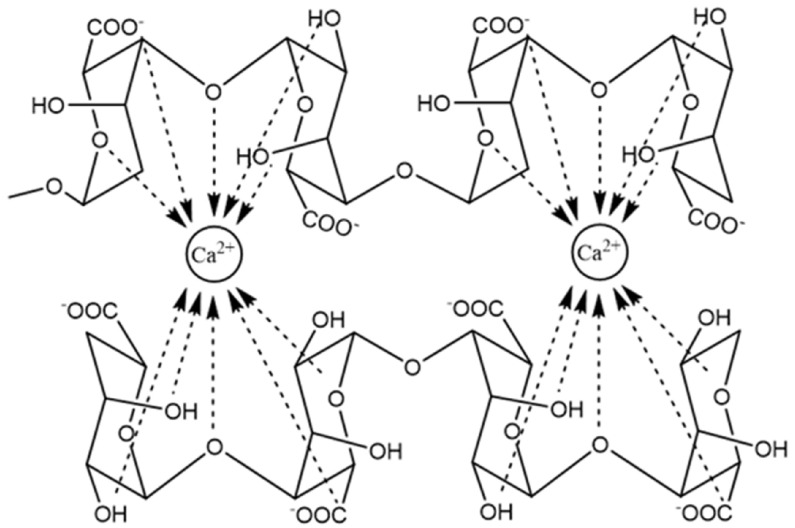
The junction zone in the egg-box model of calcium alginate gel. Reproduced from [88].

The molecule of alginate (Figure 2) is linear and consist of two types of monosaccharide moieties, i.e., D-mannuronic (M) and L-guluronic (G) acids connected with 1,4-glycosidic bonds. Both residue types are organized in homopolymeric blocks consisting of M or G units. In alginate structure, there are also heteropolymeric blocks containing both M and G residues scattered randomly. It is noteworthy that the structure of the polysaccharide may depend on its source [88]. For example, in alginate obtained from *Azotobacter*, higher content of blocks composed of guluronic acid is observed, while the polymer derived from *Pseudomonas* contains no G-blocks [89]. Structural differences are associated also with different physicochemical properties. Guluronic acid residues are responsible for the interaction with divalent ions and crosslink formation. Therefore, the compounds without G-blocks form softer, less stiff gels compared with those with higher guluronic acid content [90]. Among other factors affecting the properties of alginate, acetylation degree and molecular mass should be mentioned. Alginic acid is water insoluble, while its salts with monovalent cations reveal good solubility and form stable solutions. Upon the addition of di- or trivalent cation solution, alginates form gels in a process known as ionotropic gelation. Cations interact with guluronic residues located in homopolymeric blocks and crosslink the polymer into the so-called egg-box model (Figure 3). In the case of divalent cations, each cation is connected to four G residues. The process requires no harsh conditions or potentially toxic reagents, which is an important advantage in pharmaceutical and biomedical areas. However, the obtained physical gels display relatively weak mechanical durability, and the crosslinking cations can diffuse to the surrounding media, which may lead to physical matrix erosion. In some applications, this effect is a serious drawback. Calcium alginate is a main component of currently available wound dressings [91].

Another option is strong covalent crosslinking, mostly involving carboxylate and hydroxyl residues of the polymer. The most frequently utilized crosslinking agents comprise glutaraldehyde; however, other reagents such as poly(ethylene glycol)-diamine (PEG-diamine) and adipic acid hydrazide can be used [91]. Alginate can be also crosslinked with photoinitiated reaction or enzymatically. Photoinitiators applied in the first process may also be toxic. However, with proper design, this technique is minimally invasive and can also be applied for in situ crosslinking injectable gels [93]. The products of covalent crosslinking are usually more stable and elastic, and their swelling ability can be easily tuned with the crosslinking density. 

The wide spectrum of biomedical applications of alginate results from its numerous features that make this polymer an excellent material for drug and protein delivery systems, tissue regeneration, or wound healing. It is all due to versatile and biological properties, such as biocompatibility, nonimmunogenicity, low cost, chelating ability, water solubility, and flexibility, as its properties can be easily modified by blending with other materials, crosslinking or grafting with other polymers. So far, alginate is frequently applied and investigated as a potential excipient in drug delivery, mainly in sustained-release oral dosage forms. This is related to the swelling ability displayed by the polymer, allowing for the formation of a hydrogel matrix upon contact with gastrointestinal fluids and gradual drug release as a result of diffusion and matrix erosion. It is important to note that the process is pH dependent, and alginate-based matrices can be used to deliver the active ingredients to the distal parts of the gastrointestinal tract without releasing them in the stomach. The observed effect is related to the polymer structure and the presence of acidic groups, which become ionized at pH above 4.4. As a result of electrostatic repulsion, the matrix swells and releases the drug [94]. Moreover, alginate is explored as mucoadhesive excipient, allowing for close interaction between the surface of mucous membranes and dosage forms and extended drug residence time at the administration site. This effect is also associated with the presence of negatively charged carboxylate groups and hydroxyl moieties interacting with sialic acid and sulfate residues in the mucus [15].

As it was already mentioned, alginate displays excellent biocompatibility and can be used as a vehicle for cell delivery in tissue engineering and regenerative medicine, serving as matrix-forming material to obtain three-dimensional scaffolds, supporting cell growth and slowing down their immune clearance [95]. Moreover, alginate gels can be effectively used to deliver growth factors (e.g., vascular endothelial growth factor, VEGF, or basic fibroblast growth factor, bFGF) to improve angiogenesis in the engineered construct after the implantation [96]. A quick formation of a vascular network is crucial for the delivery of oxygen and nutrients to the implanted cells and for their survival rate. Both mentioned growth factors have limited stability and short biological half-life. The application of the appropriate carrier can improve their efficiency. It is also noteworthy that alginate can be applied to obtain injectable in situ gelling systems [97] or can be utilized as a bioink component in 3D printing with embedded cells [98]. 

Alginate has also been found useful for delivering bone-forming cells or osteoinductive factors, as well as their combinations. Despite undisputable advantages of using alginate matrices in this area, including their ability to fill irregularly shaped defects, minimally invasive manner, and ease of chemical modification for ensuring controlled release, some disadvantages should be also pointed out. First, the structure formed with the use of alginate is too mechanically weak to allow load bearing in the initial stages of regeneration without fixation. Moreover, the matrix degradation must be carefully controlled in case of enabling proper tissue regeneration. In order to improve the mechanical and bioactive properties of the implant, alginate is combined with hydroxyapatite [99]. Alginate is also investigated as a scaffold-forming material in the cartilage [100], nervous system [101] and other soft tissues regeneration [102]. 

## 4. Alginate in Wound Dressings

Modern wound dressings have been developed to provide optimal conditions for wound healing instead of just covering the wound surface and acting as a mechanical barrier protecting it from contamination. The most important function of the dressing is absorbing the excessive amount of exudate without significant drying of the wound bed. Among numerous currently applied dressings, a few types can be distinguished (Table 2). Taking the physicochemical nature of particular wound dressing systems into consideration, it may be assumed that some of them are similar to each other. However, an exceptional feature of commercially available alginate dressings is their extremely high absorbing efficiency, allowing for the removal of even high amounts of exudate. In alginate dressings, usually calcium alginate in its dry form is applied, and upon contact with the wet wound bed, the fibrous matrix is rehydrated. The resulting gel matrix provides a moist environment for wound healing. An important advantage of alginate dressings is their low adherence to the wound bed and easy removal. Moreover, this type of material can be used in both clean and infected wounds. To prevent dehydration of the occurring gel, usually a secondary dressing covering alginate is necessary, and in infected wounds, the external layer should be non-occlusive [12]. Alginate presents also high biocompatibility and nontoxic character, which is particularly important in the materials applied to damaged skin. An important advantage of this polymer is its versatility, as it can be used to form many different kinds of dressing materials, including nonporous films, porous foams [103], wafers [104], nanofibers [105], micro- and nanoparticles [106,107], and hydrogels [108]. Each of the mentioned forms can display different properties and can be applied for different purposes. For example, foams have high exudate absorption capacity compared to films, while nanofibers contribute to the formation of the environment similar to the extracellular matrix, which enhances the tissue regeneration process. Moreover, foam dressings can protect the wound from maceration and provide prolonged hydration time [109]. An important advantage of alginate in general is its ability to undergo ionotropic gelation occurring in the presence of di- or trivalent cations without any harsh conditions, which is beneficial for sensitive actives incorporation. Due to its anionic character, it can also form polyelectrolyte complexes with cationic polymers, such as chitosan, which can also be employed in wound management [110]. 

Alginate can be combined also with a variety of other materials, including polymers [112], herbal extracts [113], and antimicrobials [114]. Additional polymers usually are applied in order to improve its mechanical characteristics. So far, among the marketed products, combinations with carboxymethyl cellulose are available. However, many other polymers are being investigated, as well as other various modification options, such as grafting or different crosslinking procedures. Taking into consideration the possible applications of the products obtained as a result of these modifications, it must be emphasized that blending seems to be the safest approach. In the case of any procedure involving harsh conditions and potentially harmful reagents that may stay in the product as an impurity, the potential toxicity of the obtained material should be carefully investigated. Similar concerns may arise in the case of newly synthesized compounds, such as graft copolymers. 

## 5. Alginate Wound Dressings Loaded with Inorganic Particles

Metal and metal oxide nanoparticles as antimicrobial agents in recent years have garnered increasing attention for various biomedical applications, including the prevention of wound infections. In the alarming context of growing antibiotic resistance, nanoparticles of silver, zinc, copper, gold, and their oxides, as well as titanium, magnesium, cerium oxides, are seen as a potential tool to avoid this problem [115]. In this way, they have been applied in various biopolymeric hydrogel matrices [116,117,118], including alginate materials for wound healing. Although several metal compounds can be used, research on alginate-based dressings has focused on two types of inorganic antimicrobial nanoparticles: nanocrystalline silver (further referred to as AgNP) and zinc oxide (ZnO NP).

### 5.1. Nanocrystalline Silver (AgNP)

**Figure 4:**
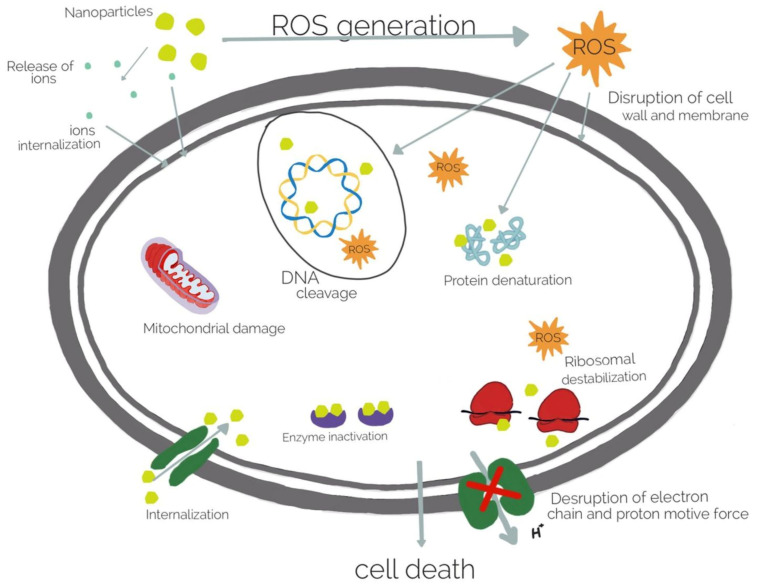
Antibacterial mechanisms of metal nanoparticles. Reprinted from [118].

Silver has been used as an antiseptic for centuries and is considered to be one of the oldest drugs known to humankind. Its antimicrobial application has mostly relied on ionic forms (silver salt solutions, colloidal silver), as it is the Ag^+^ ion that exerts bactericidal action. By interaction with negatively charged protein sites (sulfur- and phosphorus-containing groups), it disrupts the bacterial cell membrane, resulting in its death, comparatively more effective in the case of Gram-negative strains. Moreover, Ag^+^ is further capable of binding with nucleic acids, ribosome denaturation, or cytochrome inhibition once inside the cell. What is more, the generation of reactive oxygen species (ROS) can cause further damage [115,116]. A multidirectional mechanism of antibacterial activity is presented in Figure 4. Apart from bactericidal effects, silver has also been found to modulate immunological response in the wound healing process [116].

There are several commercially available alginate hydrogel dressings incorporating ionic silver (e.g., Algicell, Silvercel), which have been clinically confirmed to reduce the incidence of wound infections and improve the healing process [119,120]. However, the achievable concentration of ionic silver in wound dressing is limited by its cytotoxicity to mammalian cells. Due to this, silver nanoparticles (AgNP) are gaining popularity in various applications as an alternative, because they ensure extended, sustained release of Ag^+^ over longer periods of time without exceeding the toxic concentrations. Owing to the particle size below 100 nm, AgNP are characterized by a high surface area, which improves their contact with bacterial cell membranes. Because of this, nanocrystalline silver displays higher antimicrobial efficacy when compared with ionic forms, also in the case of wound dressings [121]. 

To the authors’ knowledge, there are no commercially available alginate-based wound healing materials that would incorporate AgNP, but research work in this field has been intensive in the last decade. It is noteworthy that Acticoat (Smith & Nephew), a nanocrystalline silver-based synthetic wound dressing, is successfully applied in wound management. 

#### 5.1.1. AgNP in Alginate-Only Matrices

Wound healing materials where alginate is the only polymeric base have been described in scientific literature, but they appear less often than alginate combinations with other polymers. One early example is a composite sponge, where AgNP were developed in situ in sodium alginate solution (by reduction with sodium borohydride), which was subsequently freeze-dried in two cycles interrupted by ionotropic Ca^2+^ crosslinking. Although the Ag-incorporating sponge successfully reduced the colonies of *S. aureus* and *K. pneumoniae* and reduced the levels proinflammatory cytokines on murine macrophage cells in vitro, it also negatively affected the viability of human fibroblasts. It must be noted, however, that the study authors did not compare different AgNP concentrations in the biocompatibility test [122].

Wet-spun calcium alginate fibers appear to be a relatively popular form for the incorporation of silver nanoparticles. Neibert et al. spun alginate solution into a calcium chloride gelling bath, followed by additional glutaraldehyde crosslinking, which permitted 20-fold hydrogel swelling (compared with 3-fold after ionotropic gelation only). The resulting fibers were immersed in AgNO_3_ solution to carry out ionic exchange between Ca^2+^ and Ag^+^ cations. With the addition of sodium borohydride, they were reduced on the fiber surface to silver nanoparticles (~10 nm). An incision wound healing study was performed on hairless mice, where AgNP-incorporating fibers at two concentrations (0.033% vs. 0.383% *w*/*w*) were compared against silver nanosuspension and blank alginate fiber. Quite surprisingly, pure AgNP suspension proved more effective in reducing inflammatory response (as determined by macrophage and neutrophil infiltration in histology samples) and ensured thicker epithelium in wound healing. Moreover, in an in vitro scratch test on 3T3 mouse fibroblast culture, AgNP alone promoted fibroblast migration and wound closure without cytotoxic effects. Therefore, in this case, alginate served only as a support and delivery platform for silver nanoparticles without observed benefits towards wound healing. The authors speculated that this could be caused by delayed Ag^+^ release from the fiber, but this was not verified experimentally [123]. Alginate microfiber was also used as a carrier by Stojkovska et al. Silver nanoparticles (10–30 nm) were synthesized electrochemically and added to sodium alginate solution, which was then extruded through a needle into a calcium chloride gelling bath. The resulting AgNP microfibers were compared against AgNP suspension in alginate solution, commercial sulfadiazine cream, and commercial calcium alginate hydrogel dressing with ionic silver. In a burn wound study on rats, all the hydrogel dressings ensured complete healing in 19 days vs. 21 days after the application of suspension or cream, which the authors attributed to the beneficial effect of Ca^2+^ ions. The nanocrystalline silver was confirmed to be more effective in wound management than the ionic form, since the same result was achieved with 2-fold lower AgNP concentration when compared with Ag^+^ in the commercial dressing [124]. 

**Figure 5:**
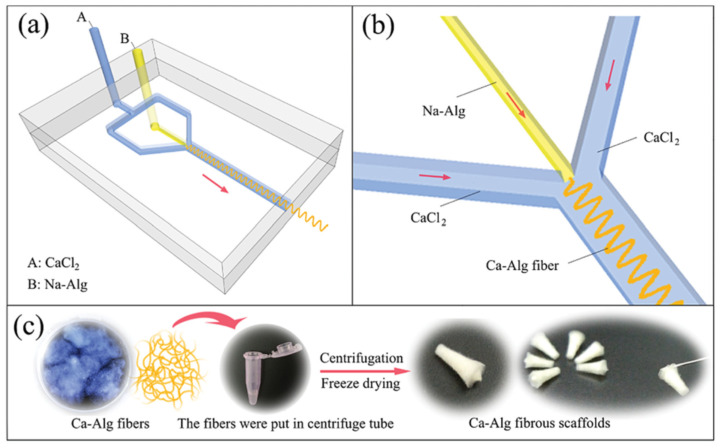
The schematic diagram shows the process of fabrication of Ca-Alg fibrous scaffolds using microfluidic spinning technology: (**a**) structure diagram of a microfluidic chip, (**b**) enlarged view of the crossing section of A and B fluids, (**c**) preparation process of Ca-Alg fibrous scaffolds. Red arrows in (**a**,**b**) correspond to the flow direction. Reprinted from [125].

An alternative microfluidic spinning process of calcium alginate fiber dressing was presented by Cai et al. AgNP were dispersed in sodium alginate solution, which was mixed in a microfluidic channel reactor with CaCl_2_ solution. A fibrous scaffold was then obtained by centrifugation and freeze-drying (Figure 5). Microbial growth tested with *S. aureus* and *E. coli* was successfully inhibited without cytotoxic effects towards L929 fibroblasts [125].

As Cai et al. pointed out, the developed microfluidic spinning method was considered as a feasible alternative to alginate electrospinning, where an addition of a suitable polymer is required for a stable fiber [125]. Indeed, nanocrystalline silver alginate dressings electrospun with polycaprolactone (PCL) or polyethylene oxide (PEO) have also been described. Hu and Lin recently developed a complex, multifunctional dressing incorporating not only silver nanoparticles, but also the gene encoding platelet-derived growth factor B (PDGF-B) to promote the wound healing process. AgNP were dispersed in PCL and PEO solution, which was then coelectrospun via a dual jet system with sodium alginate–PEO solution; the resulting fiber was ionically crosslinked with Ca^2+^. As a final step, a DNA–polyethylene imine complex was adsorbed by immersion, taking advantage of electrostatic interaction between the positively charged polyplex and negatively charged alginate. Therefore, in this dressing, AgNP were homogenously attached directly to the PCL fiber component. Silver ions were released in two stages: a quick burst in 24 h, followed by continuous, slow release over 120 h. According to the authors, such release profile is highly beneficial, ensuring a quick bactericidal effect in the initial stages of wound healing and sustained balanced exposition to Ag^+^ without cytotoxic effects. At a successful AgNP concentration of 30 mM, the proliferation of 3T3 fibroblasts was not affected. The developed dressing, which displayed antibacterial and hemostatic activity in vitro, was tested in vivo on a mouse wound model. Compared with control (gauze), alginate–PCL dressing incorporating AgNP significantly improved the wound closure rate by up to 67% in 11 days. The addition of the PDGF-B gene gave even better results, ensuring almost full wound closure (95%) at the same time point, as well as higher deposition and remodeling of collagen [126]. 

However, simpler AgNP–alginate dressing materials obtained with electrospinning have also been described. In a work by Mokhena and Luyt, AgNP were synthesized by simultaneous reduction and nanoparticle stabilization by chitosan. In this solution, an electrospun alginate (with PEO) fiber was next immersed, resulting in the fiber coating by AgNP–chitosan particles according the mechanism of polyelectrolyte complexation between alginate and chitosan. Increasing the immersion time reduced the fiber porosity and swelling capacity, on the one hand, but, on the other hand, increased the inhibition zone of G(+) and G(−) bacteria cultures. Based on the observed conductivity changes, silver was released from the dressing in a burst manner within 1 h, followed by a plateau. This behavior was sufficient to eradicate 98% and 72% of Gram-positive and Gram-negative bacterial colonies, respectively. Despite the lack of further sustained release of silver ions, no microbial growth was observed after 24 h. However, the material’s water vapor permeability of ~1500 g/m^2^/day was below the recommended values for wound dressing materials, limiting its potential use to certain types of injuries [127].

**Figure 6:**
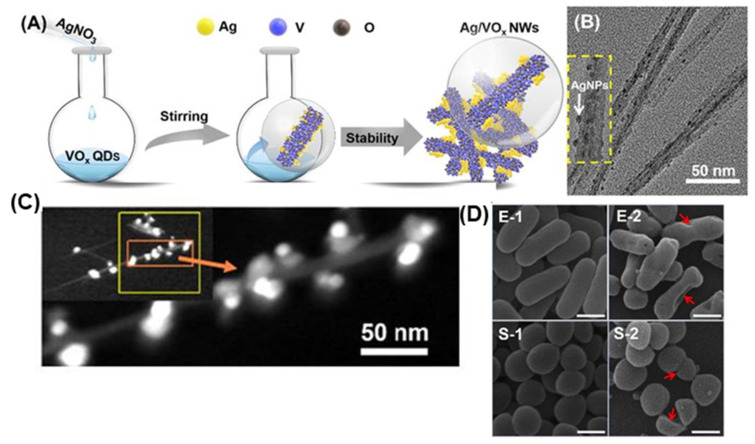
(**A**) Schematic illustration for synthesizing Ag/VOx NWs (nanowires), (**B**) TEM image of Ag/VOx nanowires, (**C**) HAADF-STEM (high angle annular dark field scanning transmission electron microscopy) of Ag/VOx NWs, (**D**) the morphology of *E. coli* and *S. aureus* characterized from SEM images without (**E-1**,**S-1**) and with Ag/VOx NWs (5 µg/mL) treatment (**E-2**,**S-2**), scale bars represent 500 nm. (Red arrows indicate bacterial cell damage). Adapted and reprinted with permission from [128].

A very interesting recent application of alginate fiber electrospinning with silver loading was described by Huang et al. as an example of combining AgNP with another inorganic nanoparticular system, namely, vanadium oxide (VO_x_) nanowires. Previously synthesized VO_x_ quantum dots were used in a reaction with AgNO_3_ as both a reducer and stabilizing agent. The resulting composite nanowires were intended to both ensure homogenous, effective binding of AgNP to the dressing and to increase the antibacterial effect while reducing the total silver content. The vanadium oxide nanowires were approximately 200 nm in length and 10 nm in width, with spherical silver nanoparticles evenly attached to the surface (Figure 6). Compared with ionic silver (AgNO_3_ solution), the AgNP/VO_x_ composite nanowires displayed higher antibacterial activity against *S. aureus* and *E. coli* at the corresponding concentrations, and their ability to damage microbial cell membranes was confirmed microscopically (Figure 6). This action was due to the composition of metal compounds, as the vanadium oxide quantum dots by themselves were not bactericidal. At the effective inhibitory concentration, the AgNP/VO_x_ nanowires were not cytotoxic towards HUVEC or HeLa cell lines.

The prepared AgNP/VO_x_ nanowires were added to alginate solution for electrospinning (with PEO); they did not alter the fiber morphology and were homogenously distributed. The nanowire-loaded fibers were then compared in vivo against blank alginate electrospun fibers and gauze as control in a full thickness wound model on rats, infected with *S. aureus* or *E. coli*. The healing process with the developed dressing was significantly faster, and the bacterial viability dropped to less than 20% (compared with 60% after the application of blank alginate and 90% in the control group). In the AgNP/VO_x_ alginate dressing treatment group, no edema was observed, and epithelium was significantly thicker, with a reduced number of inflammatory cells and more fibroblasts. However, the authors did not compare in vivo the developed material with the treatment with any other silver form [128]. 

Apart from the abovementioned work employing vanadium oxide, other inorganic nanoparticles have also been used recently as carriers to immobilize nanocrystalline silver in alginate dressing matrices. An innovative work in this topic was presented by Linhart et al. Silver ions were reduced photochemically on the surface of titanium dioxide nanoparticles, forming composite core/shell Ag/TiO_2_ nanoparticles. These were subsequently dispersed in sodium alginate solution, containing glucono-δ-lactone (GDL), calcium chloride, and one of three amino acids: glycine, serine, and arginine. Films were cast, frozen and dried. One of the study aims was to evaluate the influence of these biomimetic additives (intended to support skin regeneration) on alginate hydrogel properties. GDL served as additional a crosslinker, and amino acids chelated the Ca^2+^ ions, modifying their interaction with polymer chains. A relationship was found between hydrogen bond donor/acceptor capacity and gel packing density, its initial mass, and swelling properties. In contrast to Ag^+^ ions, the Ag/TiO_2_ loaded hydrogels did not display activity against *E. coli* in a Kirby–Bauer disc diffusion test. On the other hand, a test on a single bacterial cell confirmed its death upon contact with Ag/TiO_2_ hydrogel, with the fastest progress in the samples from which ionic silver had been removed by dialysis. It is a noteworthy experimental verification of the bactericidal mechanism of metal nanoparticles, as it was confirmed that it is due to the direct contact of the cell membrane with nanosilver, instead of Ag^+^ ions release and diffusion. Furthermore, wound healing was simulated in vitro in a scratch test on human dermal fibroblast culture (HDFa)—faster proliferation was noted in hydrogels with biomimetic additives, in positive correlation with hydrogen bonding sites [129]. A similar work was carried out by Ambrogi et al., where silver–silica composite nanoparticles were synthesized by solid-state pyrolysis from Cab-O-Sil and silver acetate. The aim of this immobilization was to avoid AgNP dispersion beyond the site of dressing application and achieve localized prolonged release of Ag^+^ for increased antibacterial activity and decreased cytotoxicity. The composite nanoparticles were dispersed in sodium alginate and glycerol solution from which a film was cast, dried, and crosslinked with calcium chloride. The presence of Ag/Cab-O-Sil increased the hydration properties of the film in simulated wound fluid. Silver ions were released from the dressing according to Higuchi kinetics, with initial burst followed by slow release to 3% in 24 h without reaching a plateau. Due to low levels of Ag^+^, the microbial growth was inhibited only in the area of direct contact with the hydrogel film. Unfortunately, the alginate hydrogels with composite silver–silica nanoparticles were found to reduce cell viability in fibroblast and keratinocyte cultures [130]. 

Apart from silver nanocomposites with other inorganic materials, AgNP have been immobilized on organic carriers in alginate wound dressings. Shin et al. partially oxidized surface hydroxyl groups to carboxyl ones in cellulose nanofibers, where Ag^+^ ions were subsequently adsorbed and reduced in situ. After the addition to sodium alginate solution, initial loose crosslinking of the polymer was performed with calcium sulfate in order to prepare an injectable hydrogel. In the next step, the material was injected to form a patch shape, with final ionic crosslinking by calcium chloride. The oxidation of cellulose nanofibers enabled higher silver loading via electrostatic adsorption, while reduction to metal nanoparticles ensured biocompatibility with 3T3 cells, in contrast to corresponding nanofibers with Ag^+^ ions. The Ag release from the dressing was slow and sustained (15% over 1 week) [131]. Another example of AgNP immobilization in nanomaterials is a study by Liang et al. on silver–polydopamine (PDA) nanocomposites in an oxidized alginate hydrogel. First, an oxidized alginate sponge was prepared by freeze-drying. Next, simultaneous dopamine polymerization and PDA adsorption on the sponge was performed, with PDA acting as a crosslinking agent. Finally, the hydrogel was immersed in silver nitrate solution, resulting in adsorption and reduction in situ to AgNP. The dressing successfully inhibited bacterial growth without hemolysis or cytotoxicity to L929 mouse fibroblasts up to 400 µg/mL, as well as shortened blood clotting time [132]. 

**Figure 7:**
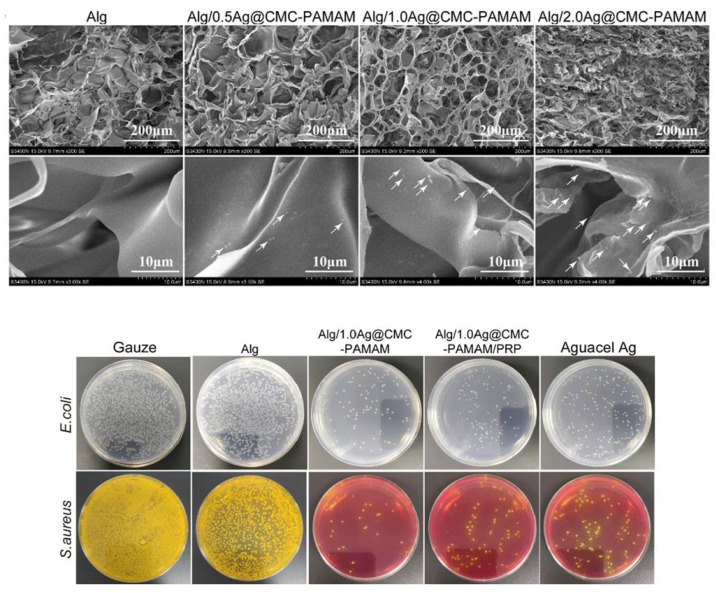
(**Top**) SEM images of the lyophilized Alg (pure alginate hydrogel), Alg/0.5Ag@CMC–PAMAM (alginate hydrogel with silver–carboxymethyl chitosan–polyamideamine composite nanoparticles), Alg/1.0Ag@CMC–PAMAM, and Alg/2.0Ag@CMC–PAMAM dressing. The white arrows point to the nanoparticles adhering to the dressing matrix. (**Bottom**) Bacterial colonies’ formation of *E. coli* and *S. aureus* from rat wounds after 3 days. Adapted and reprinted with permission from [133].

In the field of organic silver nanocomposites in alginate dressings, the most extensive work was presented by Zhou et al. Ag^+^ ions were reduced in situ and incorporated on the prepared copolymer CMC–PAMAM (carboxymethyl chitosan–grafted–polyamideamine). The composite nanoparticles were added to sodium alginate solution along with platelet-rich plasma to improve healing properties; the dressing was vacuum-dried and crosslinked with Ca^2+^. The prepared hydrogel had a homogenous, interconnected porous structure with Ag@CMC–PAMAM nanoparticles (~158 nm) adsorbed on the surface (Figure 7). Raising the nanocomposite loading in the matrix resulted in higher alginate crosslinking density, which translated to a lower swelling degree and better tensile strength and elongation properties (elasticity) of the hydrogel. The dressing proved biocompatible with L929 fibroblasts and nonhemolytic, with a water vapor transmission rate in the range of 2000–2500 g/m^2^, considered to be optimal for wound dressings. Finally, the prepared material was tested in vivo (with or without the incorporation of platelet-rich plasma) in rat wounds infected with *E. coli* and *S. aureus*. The healing rate was close between the composite dressings and commercial Aquacel Ag and better than a blank alginate hydrogel or gauze. However, based on the colony count in samples taken from the wounds, the developed Ag@CMC–PAMAM alginate dressings displayed a superior antibacterial effect over the marketed silver dressing (Figure 7, bottom). Histological analysis and the determination of factors’ expression (e.g., CD31, TNF-α, IL-6) further proved the successful healing improvement by the new materials (both for the hydrogels with and without plasma), as they ensured a lower inflammatory response, faster epithelization, and thicker epidermis with tighter connections to the dermis. Therefore, the developed Ag@CMC–PAMAM alginate dressing provided an overall multidirectionally better performance in an infected wound than an existing marketed ionic silver product [133]. 

#### 5.1.2. AgNP in Hydrogels Composed of Alginate and Other Polymers

Several wound dressings incorporating nanocrystalline silver have been prepared from blends of alginate with biopolymers (polysaccharides such as chitosan, hyaluronic acid; proteins) or synthetic polymers. 

Of the biopolymers used in blends with alginate, chitosan has proved to be the most popular one, e.g. in a freeze-dried matrix impregnated with aloe gel and AgNP [134]. Gordienko et al. developed an alginate–chitosan composite sponge incorporating silver nanoparticles (80–120 nm) synthesized from AgNO_3_ in fungal cultures, stabilized by their proteins. The dressing was prepared in several stages, starting from freezing the AgNP-containing alginate solution as an initial template. Chitosan solutions with or without CaCl_2_ were subsequently added and after a couple of thawing cycles the hydrogel was finally freeze-dried. The sponge consisted of lamellar alginate structures with tightly packed granular sites of chitosan, with the water sorption capacity increasing with the increase in alginate content. *S. aureus* and *B. cereus* growth was successfully inhibited; however, the dressing was less effective against *P. aeruginosa* [135]. In another study, hydrogel films were cast from alginate and modified hydroxytehylacryl chitosan, with or without additional ionic crosslinking by Ca^2+^. Silver particles were formed by in situ reduction in the ready film. The material was biocompatible with a Vero cell line, active against *S. aureus* and *E. coli*, and its mechanical properties were considered ‘suitable for artificial skin application’ [136]. 

An atypical combination of alginate and chitosan in wound dressing was presented by Choudhary et al., who prepared an extrudable, semisolid hydrogel on a chitosan matrix, incorporating both alginate-stabilized AgNP (20–35 nm) and nanoparticles of calcium alginate as a hemostatic agent (mean size of 1037 nm). A sustained release of silver over 24 h and activity against G(+) and G(−) bacteria was confirmed. The dressing was tested in vivo on full thickness excision wounds in a diabetic rat model. In comparison with a commercial hydrogel ointment with colloidal silver (Silverex) as positive control, the developed dressing ensured a significantly faster wound contraction rate over the whole treatment period. Collagen and tissue granulation markers (hydroxyproline and hexosamine) were also significantly elevated; revascularization and formation of homogenous and regularly distributed collagen fibers was observed as well [107]. 

Another complex multicomponent injectable hydrogel dressing based on oxidized alginate, and carboxymethylated chitosan was presented by Ma et al. Composite nanoparticles of epigallocatechin gallate (EGCG) with immobilized silver were first synthesized in a green manner from AgNO_3_ solution, with the polyphenol simultaneously serving as a reducer, AgNP carrier, antibacterial agent, and free radical scavenger. The developed Ag–EGCG composite nanoparticles were dispersed in oxidized alginate solution, together with nanoparticles of keratin, incorporated in the dressing to promote epithelialization, revascularization, and collagen deposition. Upon adding this mixture to carboxymethyl chitosan solution, Schiff reaction between the two polymers took place, crosslinking into an injectable hydrogel, to be gelled in situ upon application on wound. The formulation was subjected to extensive rheological testing, indicating self-healing gel properties; it was also confirmed to swell by 155% and retain 73% of moisture over 48 h. The hydrogel, biocompatible with L929 fibroblasts, was tested in vivo on rat wounds. However, since the authors compared the results only with saline as a negative control, and not with any alternative treatment options, it is impossible to draw conclusions on the relative efficacy of the developed dressing on wound healing [137]. 

Another popular polysaccharide used together with alginate in AgNP-containing wound dressings is hyaluronic acid. For example, sodium alginate and hyaluronic acid films were cast, dried, and gelled ionotropically (with the evaluation of different divalent cations), incorporating AgNP alone or with sulfadiazine. The hydrogels with silver nanoparticles performed better against *E. coli* and *S. aureus* than the antibiotic-loaded ones. The dressing was compared against blank sodium alginate, alginate, and hyaluronic acid hydrogels in a rat excision wound model. While there were no differences in wound area at days 7 and 21 of the treatment (full healing), the wound reduction with the dressing incorporating both AgNP and sulfadiazine was significantly higher after 14 days [138].

**Figure 8:**
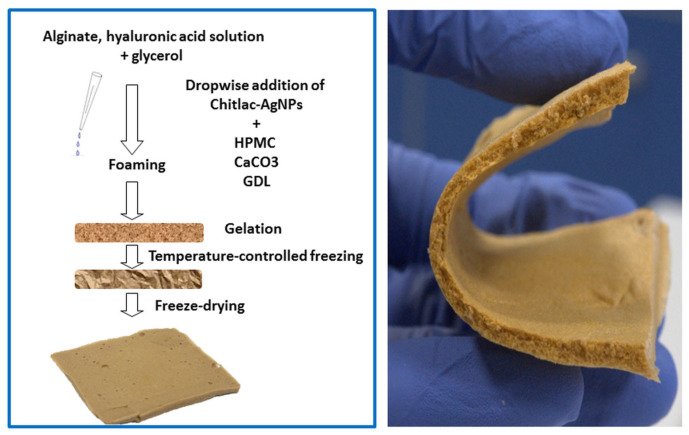
Sketch of the procedure to obtain foamed membranes containing Chitlac–AgNP (**left**) and an image of a membrane with cut edge where the internal spongy structure and the flexibility of the material can be observed (**right**). Reprinted with permission from [139].

In a work by Tarusha et al., a composite porous membrane incorporated AgNP stabilized by Chitlac (lactose-modified chitosan). The nanoparticles were dispersed in a mixture of sodium alginate, hyaluronic acid, hypromellose (as a foaming agent), and glycerol. In situ gelling was then performed with calcium carbonate and glucono-δ-lactone, followed by 30 s of foaming, further gelling, and final lyophilization. As a result, a flexible membrane was obtained (Figure 8) with a smooth surface and highly porous cross section. The dressing displayed quick swelling and very slow silver release (0.9% in 1 week), with AgNP remaining in hydrogel reservoir. The incorporation of nanoparticles did not affect the dressing’s mechanical properties or its optimal water vapor transmission rate (1920 g/m^2^/day). It is noteworthy that bactericidal activity against *S. aureus*, *S. epidermidis*, and *P. aeruginosa* was confirmed both in plankton culture and on biofilm. The dressing was also cytocompatible with human primary fibroblasts (HDFa) and keratinocytes (HaCaT). In an in vitro scratch test on monolayers of these cells cultures, wound closure was significantly improved. Moreover, in comparison with blank foam dressing, the incorporation of AgNP decreased the activity of matrix metalloproteinase (MMP), a proteolytic enzyme hindering the wound healing process. The study, therefore, confirmed the positive role of silver nanoparticles, not only for the prevention of infection, but also in supporting the wound healing process [139]. 

A very noteworthy and interesting study from the practical point of view was carried out by Catanzano et al. Ultrasmall (<1 nm) silver nanoparticles were added to a solution containing sodium alginate, hyaluronic acid, and calcium carbonate with glucono-δ-lactone, which was then cast into plates. The authors stressed that this internal gelation by CaCO_3_ and GDL of precursor hydrocolloid solution is a simple method to obtain transparent, mechanically resistant hydrogels suitable for cutting or packaging and with such simplicity can be employed in a hospital pharmacy setting. In a rarely seen angle in the literature on metal nanoparticles in alginate dressings, the researchers also discussed the different possible modes of AgNP generation or incorporation in hydrogels, indicating that the addition of previously synthesized nanoparticles is optimal, as it does not affect the gelling process and the dressing’s mechanical properties. The hydrogel’s stability was tested with the use of a modified Enslin apparatus, simulating the application on wound by wetting one side of the dressing with PBS. It was found that degradation was quicker in the presence of hyaluronic acid; the incorporation of AgNP did not affect this behavior. Another interesting, novel, and clinically relevant aspect of this work is the evaluation of the hydrogel’s antibacterial activity on real clinical isolates of multi-drug-resistant bacterial strains: methicillin-resistant *S. aureus* (MRSA), *P. aeruginosa*, *E. coli*, *A. baumannii*, and *K. pneumoniae*. It was found that the dressing was more efficient against Gram-negative bacteria, presumably due to lack of a cell wall hindering Ag^+^ diffusion towards the membrane. However, the silver nanoparticles loaded into the matrix were less effective than the corresponding concentration of pure ultrasmall AgNP, and moreover, the reduction in CFU was successful after 24 h, but not after 48 h. The authors explained this phenomenon with the influence of the hydrogel matrix: with progress in its dissolution, the interactions of Ag^+^ ions with polymer chains increase and medium viscosity is raised. According to this, they highlighted the importance of proper frequency in dressing change. Apart from antibacterial activity, the potential adsorption of human serum proteins (albumin, fibrinogen) to hydrogel matrix was also evaluated, as it could lead to the biofouling of the dressing and inflammatory reaction. The presence of hyaluronic acid reduced the adsorption, in correlation with reduced swelling. Finally, cytocompatibility and simulated healing in a scratch test were evaluated on human adipose mesenchymal stem cells. With dressings containing 10 and 50 µg of AgNP, cell viability was unaffected, while mobility and wound closure were improved [140].

Proteins have also been combined with alginate in nanocrystalline silver wound dressings. Undoubtedly, gelatin is the most popular biopolymer of this group. For instance, AgNP-loaded alginate beads were electrosprayed into a calcium chloride bath, added to gelatin solution, crosslinked with genipin, and the whole scaffold was freeze-dried. According to the authors, the developed material could be applied for wound treatment due to nontoxicity to human dermal fibroblasts and high swelling (900%) [141]. In another work, AgNP were synthesized in situ in alginate and gelatin solution by UV irradiation of AgNO_3_. Subsequently, gelatin crosslinking with glutaraldehyde was performed, followed by casting and ionotropic gelation with CaCl_2_. Silver ions were released from the hydrogel first in burst, then in a sustained manner up to 40%, depending on the calcium chloride concentration in an unexpected manner, with higher release from a more densely crosslinked matrix. The preparation was confirmed to be effective against G(+) and G(−) bacteria and biocompatible with human fibroblasts [142]. In yet another work, no additional gelling agent was used. Instead, a hydrogel was formed by mixing sodium alginate and gelatin, followed by in situ silver reduction to AgNP. The most favorable dressing with respect to consistency and flexibility was obtained from 80% alginate and 20% gelatin. In a rat excision wound study, the prepared AgNP dressing significantly reduced the wound area compared with a blank alginate–gelatin hydrogel, and provided more mature granulation tissue and reduced inflammatory response after 7 days [143]. 

Of proteins, collagen has also been used with alginate, based on the provision that it is the main component of the extracellular matrix, promotes cell proliferation and attachment, and acts hemostatically. PVP-stabilized AgNP were added to sodium alginate solution, mixed with collagen, cast into a mold, and crosslinked with calcium chloride. The antibacterial activity of the dressing against *S. aureus* and *E. coli* was confirmed with a minimal inhibitory concentration of 25 µM AgNP, well below its tested cytotoxicity threshold (50 µM) to mouse fibroblasts L929 [144]. 

**Figure 9:**
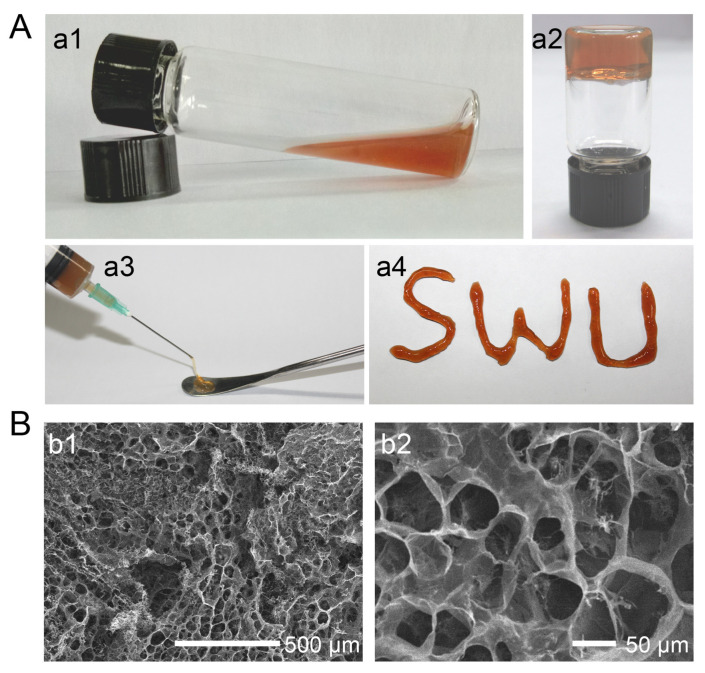
SA/Se–Ag (alginate/sericin–silver) hydrogels and SEM images. (**A**) Photos of SA/Se–Ag hydrogels without (**a1**) or with (**a2**) calcium ions. SA/Se–Ag hydrogels were injectable (**a3**) and moldable (**a4**). (**B**) Low (**b1**) and high (**b2**) magnification FESEM images of SA/Se–Ag0.2 hydrogel. Reprinted with permission from [145].

Of silver-containing alginate dressings with proteins, the most noteworthy work employed silkworm-originated sericin with beneficial effects on keratinocyte and fibroblast proliferation. By forming a semi-interpenetrating gel network (semi-IPN) of alginate with this protein, Tao et al. intended to improve the hydrogel’s adhesiveness to cells. Sodium alginate, silver nitrate, and calcium gluconate solution were prepared. Then, the addition of sericin enabled the “green” reduction of silver ions with simultaneous AgNP stabilization (15–19 nm). The resulting semi-IPN was crosslinked with Ca^2+^. The resulting hydrogel was easily injectable and moldable (Figure 9), with the swelling degree unaffected by the presence of AgNP. Silver release from the dressing was extended over 60 h without reaching a plateau. Significant activity against *E. coli*, *S. aureus*, and *P. aeruginosa* was observed with microscopically confirmed bacterial cell damage as the main mechanism. In rat wounds infected with *S. aureus*, the AgNP-loaded hydrogel resulted in significantly faster healing, achieving wound contraction of 99% vs. 81% in a blank matrix and 59% in a control group after 12 days. Moreover, only this dressing prevented the appearance of pus and inflammation symptoms, as well as promoted denser keratinocytes and angiogenesis [145]. 

Of the synthetic polymers used in silver-loaded alginate dressings, polyvinyl alcohol (PVA) appears to be applied most often. For example, its addition to extruded alginate (micro)fibers incorporating AgNP enhanced their swelling and mechanical properties (Young’s modulus, tensile stress) after rehydration [146]. In another study, Chen et al. used sodium alginate to reduce silver nitrate and stabilize the resulting AgNP, which were then mixed with alginate, carboxymethyl chitosan, and PVA solution. Hydrogel was formed by freezing and thawing, with final ionic crosslinking by Ca^2+^. In a rare approach among the reviewed studies, focus was placed on evaluating the influence of different AgNP concentrations on the composite hydrogel properties. For instance, it was found to affect the dressing’s porosity and swelling, although with no clear trend. The nanocrystalline silver also promoted PVA crystallization, resulting in better mechanical and rheological properties of the hydrogel. The dressing was favorably flexible, pliable into various forms, and resistant to strain. With an increase in AgNP content, tensile strength initially improved, but with further increase, it dropped, as the nanoparticles tended to agglomerate and hindered the formation of a hydrogel structure. The dressing displayed higher activity against *E. coli* than *S. aureus* and was cytocompatible with NIH 3T3 cells [147]. 

In another study on the improvement of mechanical properties of the dressing by combining alginate with PVA, synthesized AgNP were added to the polymers’ solution, which was freeze-thawed and gelled with calcium chloride. Moreover, 5-hydroxymethylfurfural (5-HMF) as an antioxidant and anti-inflammatory compound was added to the dressing by soaking. Increasing the proportion of alginate to PVA increased the hydrogel’s porosity, swelling, and water content, and the dressing’s compression module indicated that its mechanical stability was imparted by polyvinyl alcohol. The growth of *S. aureus* and *P. aeruginosa* was successfully inhibited, and no cytotoxicity towards L929 mouse fibroblasts was observed. In addition, pure 5-HMF was confirmed to stimulate fibroblast proliferation, migration, and collagen synthesis. The developed dressing containing AgNP and 5-HMF, either used as single components or in combination, was tested in rat wounds against a blank hydrogel. The fastest healing was observed in the AgNP+5-HMF hydrogel. Additionally, regardless of the presence of silver, the 5-hydroxymethylfurfural dressings ensured faster revascularization and formation of the epidermis [148]. 

Of synthetic polymers combined with alginate, polyvinylpyrrolidone (PVP) was also used in a dressing where both AgNO_3_ reduction to nanoparticles and polymer crosslinking were performed by gamma irradiation. The composite hydrogel was transparent and mechanically robust, with a simulated wound fluid absorption capacity of ~2000% and a water vapor transmission rate in the ideal range for wound dressings (88–278 g/m^2^/h). Even a blank alginate–PVP hydrogel was confirmed to be impermeable to bacteria and *C. albicans*, and a complete fungicidal and bactericidal effect was found to take place at a concentration of AgNP of 30 and 70–100 ppm, respectively [149]. 

### 5.2. Zinc Oxide Nanoparticles (ZnO NP)

Similar to other metal and metal oxide nanoparticles, the antimicrobial activity of ZnO is considered to rely on several mechanisms (see Figure 4): binding to cell membrane lipids and proteins, photocatalytic generation of reactive oxygen species, and possibly the release of Zn^2+^ ions and metabolism disruption inside the cell [150]. In the form of nanoparticles, bactericidal and fungicidal effects are augmented owing to the increased surface area and contact with cell membranes. Although ZnO NP are used less frequently in wound management than nanocrystalline silver [117], some research work has been carried out on its incorporation in alginate dressings, in either single or hybrid hydrogel matrices. 

Interestingly, examples of ZnO NP loaded into alginate-only dressings are scarce. However, a vital clinical work in this field was recently presented by Loera-Valencia et al. Human volunteers suffering from diabetic foot ulcer were treated either with pure calcium alginate dressing (Cutimed commercial patch) or with the same type of patch impregnated with ZnO NP. Although no significant differences were found with respect to the final assessed wound area (characterized by high variability), the difference in healing degree in favor of ZnO NP alginate dressing (75% vs. 71%) was statistically significant. The studied dressing promoted epithelialization and healthy tissue granulation. In conclusion, the clinical study demonstrated the safety and efficacy of alginate dressings loaded with zinc oxide nanoparticles [151].

**Figure 10:**
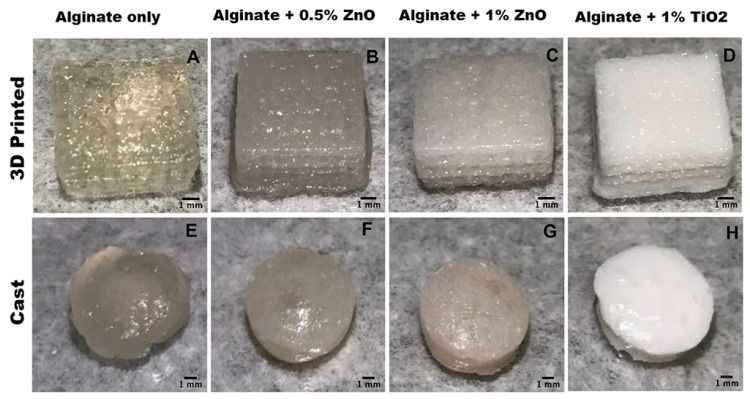
(**A**–**D**) depict 3D-printed lattice structures. (**E**–**H**) portray manually casted structures. Scale bar in all images depict 1 mm. Reprinted from [152].

In another study where alginate as the only matrix was used, Cleetus et al. developed a hydrogel with ZnO NP and compared it with an alginate hydrogel containing another type of inorganic nanoparticles, i.e., TiO_2_. Moreover, one of the research aims was to compare two methods of hydrogel production: manual casting and 3D printing, followed by ionotropic gelation by calcium chloride. The more sophisticated method ensured a more homogenous lattice structure (Figure 10) and smaller, more evenly distributed pores. This also provided better hydrogel stability owing to reduced ion diffusion through smaller pores. Three-dimensionally-printed dressings also displayed higher swelling capacity, while rheological analysis revealed that manually cast hydrogels had a significantly higher elastic modulus and were stiffer. Moreover, the incorporation of ZnO NP was observed to modify the rheological and mechanical properties as well. In contrast to a blank sample and 3D-printed alginate hydrogel with titanium dioxide, the zinc oxide nanoparticles imparted better stability to the dressing, which did not degrade in PBS over 28 days, most likely because Zn^2+^ ions had contributed to crosslinking. The alginate matrix with 0.5–1% ZnO NP showed higher activity against *S. epidermidis* not only over TiO_2_ hydrogel, but also over erythromycin. At the same time, there were no cytotoxic effects towards fibroblasts [152].

ZnO NP were also used together with another type of inorganic nanoparticles, namely, calcium phosphate (CaP), in a study by Rahman et al., where the dressing’s matrix was formed from alginate and chitosan (1:2). The synthesized nanoparticles were dispersed in alginate–chitosan solution, and subsequently, sodium tripolyphosphate as a crosslinker was added. The composite blend was finally precipitated by NaOH and freeze-dried. The dressing was active against *E. coli* and less against *S. enterica*. Not only did it prove biocompatible with baby hamster kidney fibroblasts (BHK-21) and Vero cell lines, but also in the former culture it enhanced viability when compared with the control, demonstrating the beneficial effect on fibroblast proliferation. In full thickness excision wound on mice, the biocomposite dressing markedly improved the closure rate over gauze as control, with full healing after 10 days and visible reepithelization and collagen deposition. Nevertheless, the authors did not carry out in vivo comparison with any other more advanced treatment option [153]. 

As evidenced by the abovementioned work, and similar to nanocrystalline silver dressings, chitosan is a popular complimentary polymer in alginate hydrogels incorporating ZnO NP. In a study by Nozari et al., ZnO NP-loaded film dressings were prepared based on chitosan and a gelatin matrix with the addition of either alginate or bentonite, without crosslinking agents. This difference in composition translated to several differences in the physicochemical properties of the material. The hydrogels prepared with alginate were more porous, more fragile, and less flexible, with a lower swelling capacity. While all the dressings incorporating zinc oxide were antibacterial, the alginate sample performed better against *S. aureus*, while bentonite against *P. aeruginosa*. Both were biocompatible with L929 fibroblasts, but in a burn wound study on rats, the chitosan–gelatin–bentonite composite provided better healing, ensuring complete coverage with new epithelium and hair follicle formation [154].

**Figure 11:**
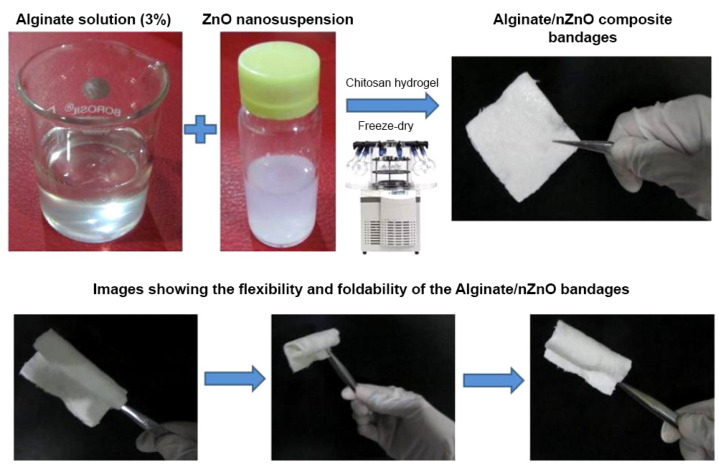
Photographical representation of the preparation of alginate hydrogel/zinc oxide nanoparticle (nZnO) composite bandages. Reprinted from [155].

Mohandas et al. added reprecipitated chitosan “pellet” to sodium alginate solution as a crosslinker for alginate hydrogel strengthening. The sample was then loaded with zinc oxide nanoparticles and freeze-dried to obtain a porous, flexible composite bandage (Figure 11). At a loading above 0.5%, ZnO NP were found to significantly decrease the swelling degree without affecting the dressing degradation kinetics in PBS containing lysozyme. At 0.75%–1% loading, the ZnO NP dressing was more effective at inhibiting the growth of *S. aureus*, *E. coli*, and *C. albicans* than a blank hydrogel and commercial alginate dressing (Kaltostat). Moreover, above 5%, the developed composite bandage was also bactericidal towards MRSA. Despite this antimicrobial potential, its applicability would clearly be limited by the observed cytotoxicity against human dermal fibroblasts (HDF), as even above a relatively low threshold concentration of 0.25%, ZnO NP cell viability was decreased to 40%–60%. Cell attachment to a nanoparticle-containing composite bandage was also reduced in comparison with a blank hydrogel. Nevertheless, in an ex vivo test on porcine ear skin, the developed dressing improved keratinocyte migration and proliferation with better epithelialization. At the same time, in a blood clotting test, the ZnO NP bandages provided better hemostasis than a blank hydrogel and commercial alginate dressing [155].

Alginate dressings containing zinc oxide nanoparticles have also been developed with chitosan derivatives. Zhang et al. dispersed synthesized ZnO NP in oxidized alginate solution, which was then crosslinked based on Schiff reaction with chitosan oligosaccharide. As the authors point out, the oligosaccharide has antimicrobial and wound healing properties, but does not form a hydrogel on its own, and due to this, supporting it with alginate is necessary. The incorporation of ZnO NP improved the mechanical properties of the dressing without affecting swelling or degradation; the water vapor transmission rate was similar to the value characterizing a commercial alginate dressing (Coloplast). An extended release of Zn^2+^ ions ensured the long-term antimicrobial effect of the developed dressing, which was efficient against G(+), G(−) bacteria and yeast. The hydrogel was biocompatible with blood (no hemolysis), NIH 3T3 fibroblasts, and human kidney epithelial cells 293T. In second-degree scald wound on rats, the healing rate after the treatment with the developed alginate–oligosaccharide–ZnO NP dressing was significantly higher than for a blank hydrogel and silver sulfadiazine. Histological evaluation revealed increased fibroblast proliferation and collagen deposition, as well as the formation of vessels, hair follicles, and sebaceous glands [156]. 

Arshad et al. compared a ZnO NP–loaded chitosan–alginate porous bandage with the version of the dressing prepared from modified, thiolated chitosan (TCS) with alginate. The polymer thiolation favorably changed several characteristics of the material. While both bandages presented a ‘fibrous, sheet-like structure’, the TCS one was more flexible with higher tensile strength. Simultaneously, the porosity and swelling degree were also elevated. Zinc oxide nanoparticles released from the thiolated bandage were slower and more sustained, reaching 70% after 72 h, with a difference in process kinetics (Korsmeyer–Peppas model, as opposed to first-order release from unmodified chitosan–alginate dressing). In a test of human blood coagulation, the TCS demonstrated better hemostatic properties. The authors also used a custom method to evaluate the mucoadhesive potential of both bandages, which were applied to the surface of goat skin samples attached to a disintegration tester assembly rack. During the dipping in simulated wound fluid (pH 7.4), the regular chitosan–alginate bandage remained in place for 6 h, while the superior swelling of the thiolated version ensured mucoadhesiveness for 72 h. The wound healing rate in mice was markedly improved not only over a commercial bandage, but also the chitosan–alginate–ZnO NP dressing, ensuring faster epidermal regeneration [157]. 

An interesting work in this area was presented by Zhu et al., who applied composite nanoparticles of metallic zinc-doped bioactive glass (Zn-BG) instead of the typical nanosized zinc oxide. As a material known from tissue engineering, bioactive glass was considered in the study to provide antibacterial synergy and stimulate the production of growth factors. The composite Zn-BG nanoparticles were obtained by microemulsion templating, and they were characterized by a regular, spherical porous structure with a size of 50–100 nm. They were dispersed in the solution of succinylated chitosan, which then formed an injectable hydrogel upon the addition of oxidized alginate. The rheological analysis revealed an elastic behavior of the hydrogel, increased with the incorporation of Zn-BG; on the other hand, the nanoparticles did not affect the swelling or pH-dependent hydrogel degradation. The developed formulation was effective against *E. coli* and *S. aureus*, with 100% bactericidal effect after 2 h. The composite hydrogel was not only biocompatible with L929 fibroblasts, but it also promoted cell adhesion and proliferation on the gel surface and in free spaces, confirming the rationale for the use of bioactive glass. In an in vivo test of full thickness wound healing on rats, four treatments were compared: blank succinyl chitosan-oxidized alginate hydrogel, hydrogel with the addition of the epidermal growth factor (EGF) without nanoparticles, Zn-BG hydrogel, and finally, Zn-BG hydrogel with EGF. No signs of wound infection were observed with any of the dressings, and after 6 days, fibroblasts appeared, as opposed to gauze control. Regarding the wound closure rate, the Zn-BG loaded hydrogel performed better than the EGF-loaded one, but a fastest healing was recorded for the synergistic composite dressing with both the nanoparticles and EGF (95% in 12 days). This was confirmed by histological and immunohistochemical analysis: the Zn BG-EGF hydrogel provided angiogenesis after 6 days, as well as increased collagen and myofibril deposition and reduced IL-6 level and gland formation after 12 days [158]. 

Of natural polymers other than chitosan and its modifications, gum acacia has also been used in alginate dressing containing zinc oxide nanoparticles. For example, in one study, ovoid hydrogel nanoparticles (95 nm) loaded with ZnO NP (5%) were prepared from alginate and gum acacia by crosslinking with glutaraldehyde and pelleting by centrifugation. Compared with pure zinc oxide nanoparticles, they were capable of better inhibition of *B. cereus*, but not of *P. aeruginosa*. On the other hand, the hydrogel promoted cell proliferation and monolayer confluency in a scratch test on fibroblast culture [159]. The ZnO NP–loaded alginate–gum acacia hydrogels were tested in vivo in a follow-up study on full thickness excision wounds in rabbits. Both the composite hydrogel and pure ZnO NP accelerated healing when compared with a commercial topical ointment in a concentration-dependent manner. The dressing promoted the formation of granulation tissue, collagen deposition, and re-epithelialization. Zinc oxide nanoparticles were observed to surround fibroblasts in the dermis, but not epidermis. Moreover, their capability to penetrate into the cytoplasm was noted [160]. 

The use of synthetic polymers in alginate dressings containing ZnO NP is less common than in dressings with nanocrystalline silver. One such example is poloxamer, which was applied in an antibacterial and hemostatic alginate gel loaded with zinc oxide nanoparticles, norfloxacin, and thymol [161]. In another study, the polymers’ combination was further enriched with mastic gum and crosslinked with calcium chloride, followed by freeze-drying. Different antimicrobial agents were incorporated either alone or at selected combinations: ZnO NP, AgNP, ciprofloxacin, or ampicillin. The main finding of the study was the observation that only the combination of zinc oxide nanoparticles and ciprofloxacin was effective against various strains of G(+) and G(−) bacteria (*E. faecalis*, *P. mirabilis*, *P. vulgaris*, *S. aureus*), while the incorporation of ZnO NP together with ampicillin did not improve the antimicrobial action. Interestingly, the joint use of zinc oxide and silver nanoparticles in the dressing did not provide significant bactericidal activity, which prompted the authors to conclude that metal nanoparticles may be antagonistic [162]. 

Apart from this, synthetic polymers have been used for electrospun dressings incorporating ZnO NP. In one of the earlier works, PVA and alginate (1:1) solution loaded with zinc oxide nanoparticles was electrospun without additional polymers and crosslinked with glutaraldehyde and calcium chloride. The antibacterial effect was concentration dependent. Up to 2% of nanoparticles, L929 fibroblasts displayed good adhesion and spreading on the electrospun mat, but cytotoxicity was noted above this value [163]. Electrospun nonwoven mats or membranes were also the subject of a work by Dodero et al., who remarked that while such research is abundant for silver nanoparticles, it is a less explored area for zinc oxide nanoparticles [164]. In this way, ZnO NP–loaded fiber membranes were prepared from alginate and PEO and crosslinked with strontium ions. Compared with blank mats, the incorporation of homogenously dispersed nanoparticles did not alter the uniform morphology of the nanofibers and, moreover, endowed them with better mechanical properties. The authors also remarked that the electrospun mat’s Young’s modulus, tensile strength, and storage modulus were close to those characterizing the skin. The addition of hydrophobic ZnO NP also decreased the dressing’s vapor permeability. Compared with a commercial collagen matrix, the electrospun alginate–PEO mat with nanoparticles promoted L929 fibroblast adhesion to a lesser degree but, in turn, better cell adhesion of HaCaT keratinocytes. Therefore, despite the use of Sr^2+^ ions for crosslinking, the dressing proved biocompatible. Moreover, an advantage over a collagen matrix was demonstrated, since the patch did not adsorb serum proteins [164]. As the developed alginate mat was found to be insufficiently thick and excessively prone to water uptake, the process was further modified by the addition of an electrospun polycaprolactone (PCL) layer, enabling low moisture content with simultaneous high vapor transmission [165]. 

### 5.3. Other Inorganic Nanoparticles 

Although various metals and metal oxides may be used for antimicrobial action, in alginate dressings, nanocrystalline silver and zinc oxide have decidedly dominated. Other types of inorganic nanoparticles have been used in a supporting role, often forming nanocomposites with Ag or ZnO, as reviewed in previous sections: vanadium oxide [128], titanium dioxide [129,152], silica [130], and calcium phosphate [153]. 

One recent exception employing other metal nanoparticles is the work by Gutierrez et al. on 3D-printed alginate hydrogels loaded with copper nanoparticles (Cu NP). Various crosslinking and copper nitrate reduction modes were tested. The optimal method consisted of the preparation of alginate solution with cellulose nanofibers (30%), followed by initial crosslinking with Ca^2+^. Ionic exchange with Cu^2+^ was then performed by the addition of copper nitrate. The hydrogel was subjected to 3D printing into a patch shape, and finally, the reduction of copper ions into Cu NP took place with sodium borohydride. The addition of cellulose nanofibers was proved to improve printing resolution, enable the crosslinking of the ready scaffold (instead of each layer), and prevent its shrinkage. The effects of concentrations of alginate, copper nitrate, and reducing agent were evaluated in terms of patch morphology, with 4% of the polymer required for a stable structure and 5 mM of sodium borohydride necessary to avoid collapse while maintaining complete Cu NP generation. The antibacterial effect against *E. coli* and *S. aureus* was found to increase with copper concentration [166].

## 6. Alginate Wound Dressings with Antibiotic and Antiseptic-Loaded Nanoparticles 

Wound healing is a complex and multistage process affected by numerous factors, including underlying diseases, the nature and size of the wound, blood supply, immune response, nutritional state, and also temperature, moisture, and sustained pressure. The most common challenge associated with abnormalities in the wound healing process is infection. Pathological microbial growth can occur particularly in immunocompromised patients with comorbidities, such as diabetes or peripheral vascular disease, and is one of the causes of impaired healing and chronic wound development [45,167,168]. It is obvious that both infection prevention and treatment are important to avoid complications related to chronic wound development. Taking into consideration modern wound dressings, different antimicrobial agents can be applied as additional components of wound management materials, in order to improve and modify the properties of the matrix polymer. So far, the most popularly utilized material is silver, known for its excellent antibacterial potential. Usually, the materials are impregnated with inorganic salts; however, as it was already demonstrated, the possibility to apply various silver nanoforms is extensively investigated. Among other antimicrobial agents, organic antiseptics and antibiotics can be listed. The first class is characterized by a wide spectrum of activity; however, usually, antiseptic agents can reveal some toxicity to host tissues. Antibiotics usually target specific microorganisms, but their application may be associated with the development of bacterial resistance. It must be also emphasized that the recommendations regarding antibiotic use are restricted only to infected wounds [45]. Nevertheless, studies regarding the development of novel wound dressings with enhanced antimicrobial properties are ongoing, even though the interest in the materials loaded with antibiotics is not as intense as in the case of silver nanoparticles. 

Alzarea et al. [169] proposed wound dressing composed by arabinoxylan (AX) and sodium alginate (SA) loaded with gentamicin sulfate (GS). Films for the investigation were prepared by the solvent casting technique, and different amounts of AX and SA were used with glycerol added as the plasticizer. The presence of the glycerol was significant, and films without this ingredient could not be used because of insufficient flexibility. All prepared films were transparent, smooth, flexible, and free from air bubbles. There was also no significant difference in physical appearance between gentamycin sulfate-loaded and blank films of the same concentration. The study of the mechanical properties showed that tensile strengths (TS) of the films ranged between 2.31 and 2.75 MPa, while the percent elongations at break (% EAB) varied from 54% to 67%. Additionally, the TS of the films increased, and EAB decreased with increasing arabinoxylan concentration in the films [169]. Moreover, the tensile strengths of the GS-loaded films were slightly higher than that of blank films. Authors concluded that intermolecular interactions between AX and SA resulted in the increased stiffness of the films and decreased flexibility. In order to evaluate the possibility of film application in wound management, an environment similar to exuding wounds was employed. Gelatin solution was allowed to solidify on the plate, pieces of films were placed over solidified gelatin, and the expansion of the films was measured. The expansion profile of blank films showed that during the first hour of contact with solidified gelatin, films hydrated (uptake water) and expanded rapidly. After that, the expansion of the blank films slowed down. A similar observation was performed for the expansion of gentamycin-loaded films. Moreover, all blank films maintained their shape after 24 h, and a higher expansion was observed for the film with the greater SA content. In the release study, approximately 40% of drug was released during the first hour of the investigation, and the GS release became slower after that. The equilibrium was reached by 24 h. Finally, the antibacterial effect of the films was investigated. It was demonstrated that GS-loaded films exhibited significant antibacterial effects against both Gram-positive (*S. aureus*) and Gram-negative (*E. coli* and *P. aeruginosa*) bacteria. The areas of growth of inhibition zones for investigated films were slightly higher than for the positive control [169].

Shahzad et al. [170] prepared alginate and pectin films loaded with cefazolin nanoparticles. Nanoparticles were prepared with the use of chitosan, and the main aim for antibiotic encapsulation was to prevent the burst drug release effect. The polymer matrix composed of alginate and pectin was obtained in the ionotropic crosslinking process. As a result, flexible mucoadhesive films, swelling upon contact with simulated wound fluid, were obtained. It was found that the crosslinking density decreased the swelling ability of the product. The drug was released from nanoparticle-based formulation in a sustained manner compared with free cefazolin. The analyzed products were also tested for *S. aureus* growth inhibition. Interestingly, it was shown that nanoparticles with antibiotic embedded in a polymer matrix were more efficient than nanoparticles alone. 

An interesting study was proposed by Reczyńska-Kolman et al. [171], who used antibacterial peptides as a promising alternative for traditional antibiotics in the composition of a polysaccharide film. The aim of the research was to develop a composite wound dressing based on gellan gum (GG) and a mixture of gellan gum and alginate (GG/Alg), containing lipid nanoparticles (NP) loaded with the antibacterial peptide nisin (NSN). Stearic acid was used as the lipid ingredient for the lipid nanoparticle formulation, the double emulsification/solvent evaporation method was applied, and received nanoparticles were spherical in shape with an average particle size of around 300 nm. Additionally, the presence of NSN did not influence the particle shape. Neither irregular particles nor nisin crystals were observed. Nisin-loaded nanoparticles (NP-NSN) were further used for the fabrication of wound dressings. Gellan gum and a mixture of GG and alginate at a 1:1 mass ratio (GG/Alg) were used as a composite matrix, while NSN or NP-NSN were added to append antibacterial properties. All prepared wound dressings were highly porous with a high swelling capacity. They were evaluated for the absorption of wound exudate in a simulated environment. The swelling capacity of the samples was evaluated via immersion in PBS at 37 °C for up to 48 h. As it was demonstrated, GG/Alg-based materials were able to absorb higher amount of buffer within the first 30 min of incubation than GG alone. The swelling capacity of GG–nisine and GG–nisine nanoparticles remained at roughly the same level throughout the whole experiment, while a significant decrease in swelling was observed for GG/Alg–nisine and GG/Alg–nisine nanoparticles. Additionally, the cytotoxic experiment showed that NSN-loaded stearic-acid-based nanoparticles were cytocompatible with L929 fibroblasts. Significant differences in nisine release profiles were observed between GG and GG/Alg dressings. In the case of GG, more than 50% of NSN was released from the system in the first 30 min of incubation, while it was only 30% for GG/Alg. More than 80% of NSN was released within 8 h for GG composites, while for GG/Alg, the amount did not reach 40%. The release study showed that the encapsulation of NSN within lipid nanoparticles significantly slowed down nisine release from GG-based samples for up to 24 h. No significant differences in NSN release profiles were observed between GG/Alg–nisine and GG/Alg–nisine nanoparticles. The antibacterial efficacy of prepared composite wound dressings was tested in contact with *S. pyogenes.* The results showed that the most effective antimicrobial activity against Gram-positive bacteria was observed for GG–nisine nanoparticles, while in GG/Alg dressing, it was decreased by interactions between nisine and alginate, leading to NSN retention within the hydrogel matrix. 

## 7. Alginate Wound Dressings with Other Nanoparticles 

Apart from inorganic nanoparticles introducing antibacterial features to the dressing material and organic particles loaded with antibiotic and antiseptic agents, there are also studies involving other nanoparticulate systems embedded in alginate matrices that can be useful in wound management. As can be concluded from the following examples, most of them employ different polymers as nanoparticle-matrix-forming agents; however, the active ingredient can also have a nanocrystalline form. Among the applied polymers, both biodegradable and nondegradable ones are used. Taking into consideration the active ingredients incorporated in nanoparticles, the most interesting research directions include growth factors, antioxidants, and anti-inflammatory agents as potential wound healing enhancers. 

Fan et al. [172], in their studies, emphasized the role of reactive oxygen species (ROS) in wound healing. An excessive and sustained amount of ROS impairs the process of repairing the tissue; on the other hand, ROS act as a healing promoter and, in small amounts, play a role in the physiological response. The aim of the study was to accelerate wound healing by the manipulation of the ROS level, using a topical antioxidant. The drug employed in this task was edaravone (EDA), a free radical scavenger currently used in the treatment of cerebral infarction. Due to the drug’s poor solubility and stability, edaravone-loaded Eudragit^®^ nanoparticles (EDA-NP) were obtained to minimize these limitations. Nanoparticles were made using a solvent displacement and evaporation method, and then incorporated to an alginate hydrogel matrix (EDA-NP-gel). In vitro studies showed sustained release of the drug from both EDA-NP and EDA-NP-gel formulations. In vivo tests were performed on diabetic mice with round wounds on the back. The investigated formulations were applied on the wounds for 6 days, and the healing was observed for 13 days. The study showed that low-dose EDA-NP-gel is able to promote wound healing in diabetic mice, leading to similar healing as in healthy mice. The mechanism of that action is probably downregulating of the ROS level. However, another outcome is that a high dose of EDA in alginate gel impairs repairing the tissue because of very low ROS amount. Interestingly, EDA-NP-gel used in normal mice resulted in impeded healing. 

Another approach to diabetic wound was presented by Atia et al. [173]. Alginate–gelatin wafers loaded with diosmin nanocrystals were prepared, and later tested on an animal model compared with other formulations. Diosmin is a flavonoid with antiulcer and antioxidant properties, and the form of wafer was chosen to provide longer residence on the wound. In vivo studies included 12 diabetic rats, each with three ulcers on the back, divided into groups and treated with previously selected formulations comprising differently prepared diosmin-loaded wafer and gel, as well as corresponding placebo formulations. The animals were being observed for 10 days, during which wound closure and ulcer area were evaluated. The study showed that the wound closure was higher in the formulations with nanocrystals. This leads to the conclusion that reducing the size of the diosmin particles may improve the efficiency of treatment. Moreover, wound healing was more effective in case of wafers than in gels. Wafers were able to stay on the ulcer for a longer time due to its better adherence, porosity, and mucoadhesive properties, while the main inconvenience connected with the gel was fast absorbing the exudation from the wound and losing rheological properties, which led to the detachment. Additionally, the physiological process of crust formation on the wound was hindering the gel penetration. Worth mentioning is that all diosmin wafer formulations showed better healing properties compared with placebo wafer [173].

Both studies of Fan et al. and Atia et al. emphasize that alginate-based materials loaded with drugs in nanoscale may improve wound healing in diabetic animals after topical administration.

**Figure 12:**
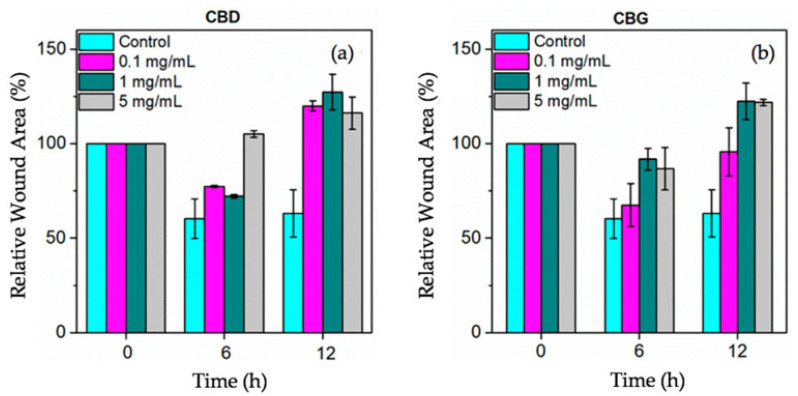
Relative wound area calculated by the in vitro wound healing assay for (**a**) CBD and (**b**) CBG nanoparticles in various concentrations (0.1, 1, or 5 mg/mL). Reprinted from [174].

Alginate wound dressing made by using a modern manufacturing technique was presented by Monou et al. [174]. Dressings contained nanoparticles loaded with cannabinoids, such as cannabidiol (CBD) and cannabigerol (CBG). Both cannabinoids demonstrate anti-inflammatory and antioxidative effects but also have low solubility in water and display instability issues caused by light and temperature. Due to the aforementioned limitations, nanoparticles with poloxamer F127 (PF127) were developed by two methods based on sonication to allow the topical administration of cannabinoids. 3D-printable inks were obtained by mixing nanoparticle suspensions with sodium alginate solution and calcium chloride solution. Printed films were also consolidated using calcium chloride solution. An in vitro study showed sustained release from formulations containing 4, 8, and 12 mg/mL CBD or CBG particles. Interestingly, it resulted with different release profiles. In the case of CBG, zero order profile was observed, while CBD profile was fitted to the Korsmeyer–Peppas model. The influence of cannabinoids’ particles on cell viability was investigated during the MTT assay. It turned out that an ether concentration of 1 mg/mL or 5 mg/mL exerted an adverse effect on cell viability. Additionally, the time of exposition was an important factor. Although CBD and CBG particles at a concentration of 0.1 mg/mL were found to be nontoxic after 24 h, the cytotoxic effect was noticed in CBD after 48 h of exposure. This indicates a dependence of cytotoxicity on both the time of the exposure and the concentration of the nanoparticles. Furthermore, that result is in agreement with the cell scratch assay performed for the same concentrations as in the MTT assay (Figure 12). 

The wounds simulated on HaCaT cells were being observed for 12 h, with 6 h intervals, and the % of wound closure was calculated. After 6 h, the wound area decreased in all samples with CBG nanoparticles and in two samples with lower CBD concentration. It is worth mentioning that none of formulations provided a significant decrease in the wound area compared with the control. Additionally, in both groups after 12 h the wound surface extended compared with 6 h exposition. Although the desired sustained release profiles were observed and antibacterial activity on selected bacterial strains were confirmed, both formulations did not demonstrate a strong wound healing effect, whereas CBG formulation seemed to be more safe for tested cells [174].

Another natural compound for wound dressing was employed by Guadarrama et al. [175]. Curcumin is known as an antimicrobial, antifungal, and anti-inflammatory ingredient. The abovementioned properties make it suitable for promoting wound healing. According to the low solubility of the active ingredient, polycaprolactone nanoparticles containing curcumin were obtained, using the emulsification–diffusion method. Subsequently, different film formulations were prepared by a solvent casting method. Sodium alginate was mixed with either polyvinyl alcohol (PVA) or polyvinylpyrrolidone (PVP), as well as plasticizers. The membrane containing sodium alginate, PVP, and propylene glycol showed the best mechanical and swelling properties and was selected to develop further film with curcumin nanoparticles. An in vitro release study of curcumin dispersion, curcumin nanoparticles (CNp), and alginate CNp matrix (CNp-M4) showed gradual release for both CNp and CNp-M4 samples. Interestingly, CNp formulation released up to 60% of drug in 48 h, with one low burst effect, while the membrane provided higher, 80% release in the same time and no significant burst effect. According to the authors, this may be explained by the interactions between nanoparticles and other matrix ingredients. The distribution of the drug and CNp was investigated ex vivo on porcine skin. Due to its low solubility in water, the drug was found mostly in superficial layers. CNp noticeably promoted reaching deeper layers of the skin. CNp released from the matrix were less likely to reach the systemic (receptor compartment), probably due to the absence of liquid medium, while in CNp dispersion, water was present. This may change in the presence of exudate from the ulcer. An in vivo permeation assay was performed on volunteers using a tape-stripping technique for all three abovementioned formulations. Cumulative concentrations from 15 tapes for each formulation after 6 h treatment were investigated. Drug released from dispersion presented the highest permeation, which is expected according to a previous ex vivo study, as the tape-stripping test refers to a superficial layer of the skin. Comparing CNp and CNp-M4, curcumin concentration in tapes was lower for film formulation, because CNp had to be released from the matrix first [175]. The investigated film formulation demonstrated promising release and permeation properties. Further studies are needed to assess the healing effect on the wound.

Alginate materials might be employed not only in reducing wound surface, but also in tissue engineering. There are examples of using the growth factor to enrich an alginate dressing’s properties in this regard. Saygili et al. [176] introduced a polyacrylamide (PAAm)–alginate (Alg) double network (DN) hydrogel loaded with poly(lactic-co-glycolide) (PLGA) nanoparticles containing transforming growth factor beta-3 (TGF-β3). The aim was to provide the release of growing factor in situ in the damaged cartilage. A nanoprecipitation method was used to produce proper nanoparticles, while the hydrogels were obtained by mixing the ingredients and two-step crosslinking. The in vitro release profile for TGF-β3 nanoparticles showed biphasic course initial burst, and then sustained release of the growth factor, with over a half of drug loading released during 24 h, and over 70% during 60 days of the study. Subsequently, the prepared PAAm-Alg matrix and matrices loaded with nanoparticles (PAAm-Alg-NP and PAAm-Alg-NP- TGF-β3), did not present a cytotoxic effect on cell viability in the MTT assay, proving the biocompatibility of formulations. Additionally, the incorporation of nanoparticles to a hydrogel resulted in increased protein adsorption, which may lead to further cell attachment and promote growth. In vitro release from PAAm-Alg-NP- TGF-β3 showed a sustained release profile with a slower onset and plateau by day 21. Cumulative release of TGF- β3 reached over 50% during 60 days of the study. An in vivo study was also conducted on rats with a trochlear groove defect. The PAAm-Alg and PAAm-Alg-NP- TGF-β3 hydrogels were implanted and left for 12 weeks. Microscopic assessment showed an improvement in tissue regeneration, especially in the PAAm-Alg-NP- TGF-β3 group, and no excessive inflammation, suggesting a potential use of hydrogel as a healing-promoting implant [176].

Lin et al. [177] employed the growth factor to develop a composite hydrogel for wound healing, and enriched it with the addition of an anti-inflammatory drug. The alginate matrix was loaded with previously prepared a poly(N-isopropylacrylamide) nanogel (pNIPAM NG) carrying basic fibroblast growth factor (bFGF) and a p(N-isopropylacrylamide-co-acrylic acid) nanogel [p(NIPAM-co-AA NG)] containing diclofenac sodium (DS). The thermosensitive properties of obtained nanogels were utilized to control drug release. Diclofenac sodium was released faster at a higher temperature, because of pushing the drug outward from shrunk p(NIPAM-co-AA), while at 37 °C, diffusion was slower, over 72 h. On the other hand, at 37 °C, only a small percentage of bFGF could be released from bFGF@pNIPAM during 14 h due to the seizing action of the nanogel. On the contrary, at a lower temperature, such limitation did not appear. An MTT Assay carried out before a study on an animal model did not show toxicity of the SA/pNIPAM/p(NIPAM-co-AA) hydrogel, so it was suitable for further research. A 14-day in vivo study with a rat model was performed using the SA/bFGF@pNIPAM/DS@p(NIPAM-co-AA) hydrogel and no treatment, SA/pNIPAM/p(NIPAM-co-AA) hydrogel, and SA/DS/bFGF hydrogel as three control groups. Treated wounds were created by burning a piece of skin on the rat dorsum and removing burned tissue with surgical tools. During the first 3 days of the experiment, dressings were rewetted using warm PBS solution (37 °C). Subsequently, from fourth day, cold PBS (4 °C) was used. This procedure was intended to initiate a two-step action of dressing, stimulating SD and then bFGF delivery. The SA/bFGF@pNIPAM/DS@p(NIPAM-co-AA) hydrogel produced the best result compared to the controls. The presented hydrogel seems to be an interesting example of more complex wound dressing and stepwise delivery of two substances to promote wound healing and exert an anti-inflammatory effect [177].

**Figure 13:**
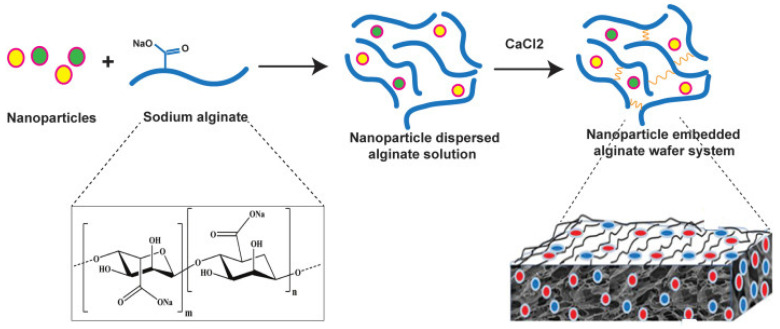
Schematic representation of the preparation of calcium alginate wafer dressing. Reprinted from [104] with permission.

Another approach was demonstrated by Vijayan et al. [104]. This study also focused on the delivery of two substances in one formulation, but in the form of a wafer. Basic fibroblast growth factor (bFGF) and vascular endothelial growth factor (VEGF) were applied together in one dressing to obtain synergistic action in wound healing. PLGA was employed to synthesize growth-factor-loaded nanoparticles by a modified solvent diffusion technique. Subsequently, a wafer was obtained by dispersing nanoparticles in sodium alginate solution, freeze-drying, and then crosslinking and once more freeze-drying the scaffold (Figure 13). 

During the release studies, a two-step release process was observed. In the first 24 h, the process was faster and corresponded to the release of the actives from the surface of the scaffold, and in the next 24 days, a sustained release was observed. In the MTT assay, all investigated formulations showed a proliferative effect on the HaCaT cell line compared with the control. Interestingly, not only a growth-factor-loaded wafer improved cell proliferation. This indicates that blank formulation is also able to provide conditions suitable for healing due to enhanced biocompatibility. To investigate wound healing properties in vivo, a study on an animal model was performed. Three groups of rats were treated with a placebo wafer, a wafer containing nanoparticles loaded with the growth factor, and PBS as the control group. The wounds were created surgically at the dorsum of the animal. Observation was carried out during 21 days, and the wound contraction was assessed. No significant difference between all groups was reported after 7 days of treatment, whereas after 14 and 21 days, it was noticeable that the wafer containing the growth factor improved healing compared with the control. According to the authors, that might be caused by increasing the concentration of the growth factor in the wound. Interestingly, in the group treated with a placebo wafer, an enhanced healing process compared with the control was also observed. This may indicate the ability of creating a proper healing environment and is in agreement with a previous MTT assay, which showed improved cell proliferation in both the placebo wafer and the loaded wafer. Although the wound healing rate for both wafers is similar, the histological examination revealed a higher amount of collagen and a higher thickness of the stratified epithelial layer in the group treated by the growth-factor-loaded wafer [104].

## 8. Wound Healing Materials with Alginate Nanoparticles

In general, nanoparticles used for wound healing can improve the distribution of the administered drug and improve its solubility. This, in turn, gives the opportunity to reduce doses, which results in the improved safety and minimized toxicity of the drug. Taking it under consideration, it can be stated that nanocomposites can be used as delivery agents and help in wound healing [178]. On the other hand, the alginate matrix may prolong the release of the incorporated active agents. Alginate nanoparticles can also be a carrier to sensitive ingredients, such as growth factors, or can act as physical stabilizers in emulsions. Alginate is one of the polymers that gain much interest in that field in recent years. Such nanomaterials can be obtained by various methods, e.g., complexation, alginate in oil emulsification, self-assembly, and complexation, complexation on the interface of emulsion droplets, and water-in-oil emulsions [179]. Apart from the previously presented examples of alginate matrices with embedded particles prepared with some other materials, alginates can also be used to form nanoparticles, alone or in blends with other polymers. Recently, Del Gaudio et al. [180] proposed a material prepared by blending high amidated pectin and high mannuronic content alginate particles for wound dressing. Particles were loaded with Ac2-26 peptide (N-terminal-derived peptide of annexin A1) using a nanospray technique. The polymeric blend enhanced the stability of the peptide and increased the release capability toward the wound cavity. Additionally, high encapsulation efficiency was achieved in that formulation (~83%). In vitro studies revealed the improvement of cell migration for blend particles, making it a suitable material for wound healing. Oliveira et al. [181] prepared a double-layer membrane for dual drug delivery to be used for wound treatment. The membrane was composed of chitosan, hydroxypropyl methylcellulose and lidocaine chloride in the first layer and of sodium alginate–polymyxin B sulfate nanoparticles in the other layer. It was characterized by a thickness of 0.01–0.02 mm and satisfying mechanical properties for potential application to the lesion. During the in vitro analytical process, an activity against *S. aureus* and *P. aeruginosa* strains and low cytotoxicity were revealed. An in vivo assay allowed visualizing the healing potential by calculating the wound retraction index and by histological analysis. The obtained results can confirm the effectiveness of the developed innovative biomaterial for tissue repair and regeneration in an animal model.

In a study presented by Wu et al. [182], composite hydrogels based on Pickering emulsions stabilized with carboxymethyl chitosan–sodium alginate (CMCS-SA) nanoparticles (NPs). Apart from the emulsion, the obtained systems contained poloxamer 407 and curcumin. The performed analyses revealed that the stability of emulsion improved with the increasing amount of NP while remaining stable at various temperatures. Furthermore, the prepared formulation showed a controlled release of incorporated curcumin. The obtained composite material exhibited activity against *S. aureus* and *E. coli* and improved the wound healing process, making it a suitable bioactive component for wound care management. 

Alginate-based nanoparticles can also be a suitable platform for diabetic and nondiabetic wound healing. Such a platform based on chitosan–alginate NP for pressure ulcer healing was proposed by Sheir et al. [183]. The authors combined the two mentioned polymers into drug-free nanosystems with positive and negative surface charges. Such nanoparticles were prepared by an ionic gelation method. NP with satisfying properties were chosen for in vivo experiments with the use of diabetic and nondiabetic rats to heal pressure ulcers. In this experiment, the rate of wound closure, histological examination, and histomorphometric assessment were used to evaluate the NP wound healing potential. Both positively and negatively charged NP exhibited a significant enhancement in wound closure rates in comparison with the control group. Furthermore, a higher quality and maturation of formed new tissue, less inflammation, and higher collagen content were also revealed. 

Another work that targeted diabetic wounds was published by Montaser et al. [184]. They prepared silver/alginate/nicotinamide nanoparticle composite for wound dressing. Sodium alginate (ALG) was used as a reducing and stabilizing agent for the preparation of silver nanoparticles (Ag-NPs). Antibacterial activity was evaluated against Gram-positive and Gram-negative bacteria and wound healing potential was evaluated with the use of burn diabetic rats. As a result, authors obtained a formulation that promoted an antibacterial effect on both Gram-positive and Gram-negative bacteria at the higher loading percent of silver colloid nanoparticles, showing the normalization of the epidermal layer in thickness and in the arrangement of cells if compared with that on both sides of the wound and also providing a fast healing character demonstrated in the histological examinations. 

Maatouk et al. [185], in their work, targeted limited matrix deposition and poor tissue repair caused by the lack of connective tissue growth factor (CTGF) and insulin-like growth factor, which can lead to the worsening of diabetic foot ulcers and results in partial or complete limb amputation. To enhance growth factor availability, they developed heparin-mimetic alginate sulfate/polycaprolactone double-emulsion nanoparticles. Such formulation was characterized by high affinity and sustained release of growth factors. The manufacturing process based on sonication resulted in the formation of particles with a size of 236 nm. Furthermore, the experiment assessing wound healing capacity in immortalized primary human adult epidermal cells showed a noncytotoxic effect after 72 h. NP containing CTGF revealed the most rapid wound enclosure compared with controls. 

## 9. Conclusions and Future Directions

Appropriate wound management is an important and challenging problem in contemporary medical care, especially in the case of chronic conditions with underlying chronic diseases. The statistics show that the need for novel, efficient, and cost-effective materials for wound healing will increase in the future with the increase in elderly population or the number of patients suffering from diabetes and other conditions, increasing the risk of non-healing wound development. On the other hand, reports referring to the efficacy of particular antimicrobials widely applied in wound management are alarming and indicate that the problem with pathogenic strain eradication may become even more challenging in the future. According to the currently available data, apart from the well-known problem of antimicrobial resistance to antibiotics, there is also evidence of resistance to silver [186,187]. Even though this issue still seems to have little clinical importance, it may be assumed that the overuse of silver-based products may result in increasing problems with non-healing wounds in the future. Moreover, the demand for novel therapeutic approaches and novel strategies for the elimination of pathogenic bacteria will most probably increase, too. 

It must be emphasized that wound healing, as a complex process with numerous different factors involved, may require individualized treatment. Different types of wounds associated with different comorbidities require different management, which is reflected by the huge variety of commercially available products. However, the research studies presented in this review indicate that this area can still be explored and novel strategies for efficient wound management can be investigated. Wound dressings can be modified in terms of polymer matrix structure and also in terms of incorporated active ingredients, accelerating the healing process. The summarized literature reports show that alginate can be easily modified by blending with other materials and incorporating various nanomedicines, including nanoparticles. It is noteworthy that its nontoxicity and biocompatible character are important advantages in biomolecule and cell delivery, which can be investigated in the development of innovative wound dressings. Alginate can also be modified chemically by crosslinking or grafting; however, in the case of newly synthesized materials, detailed toxicity studies are necessary.

Taking into consideration the general research directions explored in wound healing, growth factors and stem cell delivery seem to be among the most promising ones. It is noteworthy that there are marketed topical products with growth factors intended for the management of chronic wounds [188]. However, the available literature describing alginate-based systems loaded with nanoparticles and combined with the mentioned factors is quite scarce, and most of the studies refer to the materials with growth factors directly incorporated in the alginate matrix [189,190,191]. The published results indicate that alginate is a good material for the delivery of sensitive active agents, and the ability to form a gel in mild conditions is considered an important advantage. On the other hand, as indicated by Catanzano et al. [188], growth factors may require some additional approach to improve their stability and extend the drug release process. Therefore, alginate wound dressings with nanoparticles can be useful carriers, and it may be expected that this research direction will be further developed. 

It is noteworthy that alginate-based materials are not free from disadvantages, when wound dressing formulation is taken into consideration. Among the challenges important for the wound healing process, the low adherence of alginate is mentioned. Usually, alginate-based materials are not applied alone but are either supported by a secondary dressing, or alginate fibers are enhanced with some other material. Moreover, some side effects, including allergies, were reported in the case of an insufficient amount of moisture in the wound bed [192]. The mechanical strength of alginate can also be insufficient for the formulation of materials other than flat dressings, as more complex constructs and scaffolds can collapse [193,194]. Finally, the sterilization of alginate-based dressings can be also challenging, as literature reports indicate loss of mechanical properties and decreased swelling ability upon steam sterilization [195], impaired swelling and chemical degradation upon irradiation [196,197], and changes in mechanical characteristics as a result of supercritical carbon dioxide sterilization [198]. It is obvious that an additional nanoparticle component can potentially further complicate the process. In the context of the future application of composite alginate dressings, the selection of a proper sterilization technique and its optimization seems to be an important research direction. 

The presented nanoparticle-related studies show the enormous potential of composite materials in the development of novel wound dressings. Even though most of the studies focus on silver-loaded nanoparticles, it is obvious that these systems can be also employed to deliver many different compounds, potentially advantageous in the healing process, such as growth factors, biopolymers, or plant-derived antimicrobials. 

## Figures and Tables

**Table 1:** The examples of commercially available alginate-based wound dressings.

Brand Name	Manufacturer	Composition	Applications	Reference
Biatain^®^ Alginate	Coloplast (Humlebaek, Denmark)	Alginate (85%), CMC ^1^	Moderate to heavily exuding wounds	[20]
Biatain^®^ Alginate Ag		Alginate, CMC, ionic silver complex	Moderate to heavily exuding wounds, infected wounds	[21]
Comfeel^®^ Plus Ulcer		CMC particles, alginate (as an additive), polyurethane semipermeable film	Low to moderately exuding wounds	[22]
Kaltostat^®^	ConvaTec (Reading, UK)	Calcium sodium alginate	Moderate to heavily exuding wounds	[23]
CarboFlex^®^		Five-layer dressing with alginate absorbent layer, internal charcoal layer, and water-resistant top	Malodorous wounds	[24]
Tegaderm™ Alginate	3M™ (Saint Paul, MN, USA)	Calcium alginate	Moderate to heavily exuding wounds	[25]
Sorbalgon^®^	Hartmann (Heidenheim, Germany)	Calcium alginate	Moderate to heavily exuding wounds	[26]
Sorbalgon^®^ Ag		Calcium alginate, silver	Moderate to heavily exuding wounds, infected wounds	[27]

^1^ Carboxymethyl cellulose.

**Table 2:** Types of modern wound dressings [13,111].

Dressing Type	Properties	Examples
Semipermeable films	Transparent polyurethane with acrylic adhesive coating The wound can be monitored They are semiocclusive Provide autolytic debridement, as they keep moisture in the wound bed Enable transmission of oxygen, water vapor, and CO_2_ Recommended for shallow wounds with low exudate levels	Tegaderm (3M) Opsite (Smith & Nephew)
Semipermeable foams	Hydrophilic or hydrophobic, nonadherent They may contain adhesive borders or not Polyurethane or silicon based They can absorb moderate or high amount of exudate Allow for gas transmission but keep the moisture in the wound bed Require frequent changes They are not suitable for dry wounds	Allevyn (Smith & Nephew)
Hydrogel dressings	Insoluble hydrophilic matrix (polymethacrylates or polyvinyl pyrrolidone) Transparency allows for wound monitoring High water content (70%–90%) Cooling effect Recommended for dry wounds, necrotic wounds, pressure ulcers, and burns Exudate accumulation may lead to tissue maceration and bacterial growth Can be available as amorphous gel, impregnated gauze, or sheet hydrogel Low mechanical strength	Intrasite (Smith & Nephew) Granugel (ConvaTec)
Hydrocolloid dressings	Contain inner colloidal layer and external water-impermeable layer Contain absorbent polymers (CMC, pectin, gelatin) that form gel upon the contact with exudate Occlusive (no water and oxygen transmission) Recommended for low and moderate amounts of exudate Enable granulation and epithelialization	Granuflex (ConvaTec) Comfeel (Coloplast)
Alginate dressings	Available as fibrous nonwoven sheets and ropes Form gels upon the contact with exudate They can absorb extremely high amount of fluids (up to 20 times their weight) Can be applied with clean and infected wounds Not recommended for dry wounds Require secondary dressing preventing drying out	Kaltostat (ConvaTec) Sorbalgon (Hartmann)

## Data Availability

Not applicable.

## References

[1] Mervis J.S., Phillips T.J. (2019). Pressure Ulcers: Pathophysiology, Epidemiology, Risk Factors, and Presentation. J. Am. Acad. Dermatol..

[2] Grey J.E., Harding K.G., Enoch S. (2006). Pressure Ulcers. BMJ.

[3] Kapp S., Miller C., Santamaria N. (2018). The Quality of Life of People Who Have Chronic Wounds and Who Self-Treat. J. Clin. Nurs..

[4] Kapp S., Santamaria N. (2017). The Financial and Quality-of-Life Cost to Patients Living with a Chronic Wound in the Community. Int. Wound J..

[5] Renner R., Erfurt-Berge C. (2017). Depression and Quality of Life in Patients with Chronic Wounds: Ways to Measure Their Influence and Their Effect on Daily Life. CWCMR.

[6] Järbrink K., Ni G., Sönnergren H., Schmidtchen A., Pang C., Bajpai R., Car J. (2016). Prevalence and Incidence of Chronic Wounds and Related Complications: A Protocol for a Systematic Review. Syst. Rev..

[7] Zhao R., Liang H., Clarke E., Jackson C., Xue M. (2016). Inflammation in Chronic Wounds. Int. J. Mol. Sci..

[8] Sen C.K. (2021). Human Wound and Its Burden: Updated 2020 Compendium of Estimates. Adv. Wound Care.

[9] Nussbaum S.R., Carter M.J., Fife C.E., DaVanzo J., Haught R., Nusgart M., Cartwright D. (2018). An Economic Evaluation of the Impact, Cost, and Medicare Policy Implications of Chronic Nonhealing Wounds. Value Health.

[10] Lindholm C., Searle R. (2016). Wound Management for the 21st Century: Combining Effectiveness and Efficiency. Int. Wound J..

[11] Kim H.S., Sun X., Lee J.-H., Kim H.-W., Fu X., Leong K.W. (2019). Advanced Drug Delivery Systems and Artificial Skin Grafts for Skin Wound Healing. Adv. Drug. Deliv. Rev..

[12] Rezvani Ghomi E., Khalili S., Nouri Khorasani S., Esmaeely Neisiany R., Ramakrishna S. (2019). Wound Dressings: Current Advances and Future Directions. J. Appl. Polym. Sci..

[13] Vowden K., Vowden P. (2017). Wound Dressings: Principles and Practice. Surgery.

[14] Bi D., Yang X., Yao L., Hu Z., Li H., Xu X., Lu J. (2022). Potential Food and Nutraceutical Applications of Alginate: A Review. Mar. Drugs.

[15] Jadach B., Świetlik W., Froelich A. (2022). Sodium Alginate as a Pharmaceutical Excipient: Novel Applications of a Well-Known Polymer. J. Pharm. Sci..

[16] Kozlowska J., Prus W., Stachowiak N. (2019). Microparticles Based on Natural and Synthetic Polymers for Cosmetic Applications. Int. J. Biol. Macromol..

[17] Sahoo D.R., Biswal T. (2021). Alginate and Its Application to Tissue Engineering. SN Appl. Sci..

[18] Morgan D. (1997). Alginate Dressings: Part 1: Historical Aspects. J. Tissue Viability.

[19] Sorbsan—Sterile Calcium Aginate Wound Dressing Range. https://sorbsan.co.uk/.

[20] Biatain® Alginate. https://products.coloplast.co.uk/coloplast/wound-care/biatain-alginate/.

[21] Biatain^®^ Alginate Ag. https://products.coloplast.co.uk/coloplast/wound-care/biatain-alginate-ag/.

[22] Comfeel^®^ Plus Dressing. https://products.coloplast.co.uk/coloplast/wound-care/comfeel-plus/comfeel-plus-dressing/.

[23] KALTOSTAT^®^ Calcium Sodium Alginate Dressing. https://www.convatec.com/products/advanced-wound-care/wound-type/wound-burns/kaltostat-calcium-sodium-alginate-dressing/.

[24] CarboFlex^®^ Dressing. https://www.convatec.com/en-gb/products/advanced-wound-care/wound-type/pc-wound-pressure-ulcers-stage-3-4/carboflex-dressing/.

[25] 3M^TM^ Tegaderm^TM^ High Integrity Alginate Dressing. https://www.3m.com/3M/en_US/p/d/b5005108002/.

[26] Sorbalgon^®^. https://www.hartmann.info/en-us/our-products/wound-management/advanced-wound-care-dressings/alginates/sorbalgon®.

[27] Sorbalgon^®^ Ag. https://www.hartmann.info/en-us/our-products/wound-management/advanced-wound-care-dressings/alginates/sorbalgon®-ag.

[28] ACTICOAT Global. https://www.smith-nephew.com/en/health-care-professionals/products/advanced-wound-management/acticoat-global.

[29] Najahi-Missaoui W., Arnold R.D., Cummings B.S. (2021). Safe Nanoparticles: Are We There Yet?. Int. J. Mol. Sci..

[30] Berthet M., Gauthier Y., Lacroix C., Verrier B., Monge C. (2017). Nanoparticle-Based Dressing: The Future of Wound Treatment?. Trends Biotechnol..

[31] Tirumala M.G., Anchi P., Raja S., Rachamalla M., Godugu C. (2021). Novel Methods and Approaches for Safety Evaluation of Nanoparticle Formulations: A Focus Towards In Vitro Models and Adverse Outcome Pathways. Front. Pharmacol..

[32] Vachhrajani V., Khakhkhar P., Vachhrajani V., Khakhkhar P. (2020). Different Types of Wounds. Science of Wound Healing and Dressing Materials.

[33] McGuire L., Heffner K., Glaser R., Needleman B., Malarkey W., Dickinson S., Lemeshow S., Cook C., Muscarella P., Scott Melvin W. (2006). Pain and Wound Healing in Surgical Patients. Ann. Behav. Med..

[34] Gorin D.R., Cordts P.R., LaMorte W.W., Menzoian J.O. (1996). The Influence of Wound Geometry on the Measurement of Wound Healing Rates in Clinical Trials. J. Vasc. Surg..

[35] Percival N.J. (2002). Classification of Wounds and Their Management. Surgery.

[36] Labbé J., Caouette G. (2001). Recent Skin Injuries in Normal Children. Pediatrics.

[37] Bailey B.R., Eckerman K.F., Townsend L.W. (2003). An Analysis of a Puncture Wound Case with Medical Intervention. Radiat. Prot. Dosim..

[38] Thirupathi Kumara Raja S., Thiruselvi T., Sailakshmi G., Ganesh S., Gnanamani A. (2013). Rejoining of Cut Wounds by Engineered Gelatin–Keratin Glue. Biochim. Biophys. Acta (BBA)—Gen. Subj..

[39] Barington K., Jensen H.E. (2016). The Impact of Force on the Timing of Bruises Evaluated in a Porcine Model. J. Forensic Leg. Med..

[40] Mlambo S.S., Parkar H., Naude L., Cromarty A.D. (2022). Treatment of Acute Wounds and Injuries: Cuts, Bites, Bruises and Sprains. SA Pharm. J..

[41] Mankowitz S.L. (2017). Laceration Management. J. Emerg. Med..

[42] Lee C.J., Santos P.J.F., Vyas R.M. (2019). Epidemiology, Socioeconomic Analysis, and Specialist Involvement in Dog Bite Wounds in Adults. J. Craniofacial Surg..

[43] Rothe K., Tsokos M., Handrick W. (2015). Animal and Human Bite Wounds. Dtsch. Arztebl. Int..

[44] Dreifke M.B., Jayasuriya A.A., Jayasuriya A.C. (2015). Current Wound Healing Procedures and Potential Care. Mater. Sci. Eng. C.

[45] Han G., Ceilley R. (2017). Chronic Wound Healing: A Review of Current Management and Treatments. Adv. Ther..

[46] Kirsner R.S., Eaglstein W.H. (1993). The Wound Healing Process. Dermatol. Clin..

[47] Alhajj M., Goyal A. (2022). Physiology, Granulation Tissue. StatPearls.

[48] Sorg H., Tilkorn D.J., Hager S., Hauser J., Mirastschijski U. (2017). Skin Wound Healing: An Update on the Current Knowledge and Concepts. ESR.

[49] Weledji E. (2017). Perspectives on Wound Healing. Austin J. Surg..

[50] Guo S., DiPietro L.A. (2010). Factors Affecting Wound Healing. J. Dent. Res..

[51] Abazari M., Ghaffari A., Rashidzadeh H., Badeleh S.M., Maleki Y. (2022). A Systematic Review on Classification, Identification, and Healing Process of Burn Wound Healing. Int. J. Low. Extrem. Wounds.

[52] Gosain A., DiPietro L.A. (2004). Aging and Wound Healing. World J. Surg..

[53] Rezaie F., Momeni-Moghaddam M., Naderi-Meshkin H. (2019). Regeneration and Repair of Skin Wounds: Various Strategies for Treatment. Int. J. Low. Extrem. Wounds.

[54] Wilkins R.G., Unverdorben M. (2013). Wound Cleaning and Wound Healing: A Concise Review. Adv. Ski. Wound Care.

[55] Das A., Datta S., Roche E., Chaffee S., Jose E., Shi L., Grover K., Khanna S., Sen C.K., Roy S. (2018). Novel Mechanisms of Collagenase Santyl Ointment (CSO) in Wound Macrophage Polarization and Resolution of Wound Inflammation. Sci. Rep..

[56] Percival S.L., Mayer D., Malone M., Swanson T., Gibson D., Schultz G. (2017). Surfactants and Their Role in Wound Cleansing and Biofilm Management. J. Wound Care.

[57] Urban M.V., Rath T., Radtke C. (2019). Hydrogen Peroxide (H_2_O_2_): A Review of Its Use in Surgery. Wien. Med. Wochenschr..

[58] Lu Y.-X., Wu Y., Liang P.-F., Wu R.-C., Tian L.-Y., Mo H.-Y. (2021). Efficacy of Combination of Localized Closure, Ethacridine Lactate Dressing, and Phototherapy in Treatment of Severe Extravasation Injuries: A Case Series. World J. Clin. Cases.

[59] Bai M.-Y., Chen M.-C., Yu W.-C., Lin J.-Y. (2017). Foam Dressing Incorporating Herbal Extract: An All-Natural Dressing for Potential Use in Wound Healing. J. Bioact. Compat. Polym..

[60] Guiomar A.J., Urbano A.M. (2022). Polyhexanide-Releasing Membranes for Antimicrobial Wound Dressings: A Critical Review. Membranes.

[61] Nuutila K., Eriksson E. (2021). Moist Wound Healing with Commonly Available Dressings. Adv. Wound Care.

[62] Weller C., Weller C., Team V., Rajendran S. (2019). 4—Interactive Dressings and Their Role in Moist Wound Management. Advanced Textiles for Wound Care.

[63] Tellechea A., Leal E.C., Kafanas A., Auster M.E., Kuchibhotla S., Ostrovsky Y., Tecilazich F., Baltzis D., Zheng Y., Carvalho E. (2016). Mast Cells Regulate Wound Healing in Diabetes. Diabetes.

[64] Thelwall S., Harrington P., Sheridan E., Lamagni T. (2015). Impact of Obesity on the Risk of Wound Infection Following Surgery: Results from a Nationwide Prospective Multicentre Cohort Study in England. Clin. Microbiol. Infect..

[65] Lee E., Zhang H., Jackson J.K., Lim C.J., Chiao M. (2016). Janus Films with Stretchable and Waterproof Properties for Wound Care and Drug Delivery Applications. RSC Adv..

[66] Olsson M., Järbrink K., Divakar U., Bajpai R., Upton Z., Schmidtchen A., Car J. (2019). The Humanistic and Economic Burden of Chronic Wounds: A Systematic Review. Wound Repair. Regen..

[67] Morton L.M., Phillips T.J. (2016). Wound Healing and Treating Wounds: Differential Diagnosis and Evaluation of Chronic Wounds. J. Am. Acad. Dermatol..

[68] Rowan M.P., Cancio L.C., Elster E.A., Burmeister D.M., Rose L.F., Natesan S., Chan R.K., Christy R.J., Chung K.K. (2015). Burn Wound Healing and Treatment: Review and Advancements. Crit. Care.

[69] Kimmel H.M., Grant A., Ditata J. (2016). The Presence of Oxygen in Wound Healing. Wounds.

[70] Gompelman M., van Asten S.A.V., Peters E.J.G. (2016). Update on the Role of Infection and Biofilms in Wound Healing: Pathophysiology and Treatment. Plast. Reconstr. Surg..

[71] Mangoni M.L., McDermott A.M., Zasloff M. (2016). Antimicrobial Peptides and Wound Healing: Biological and Therapeutic Considerations. Exp. Dermatol..

[72] Sgonc R., Gruber J. (2013). Age-Related Aspects of Cutaneous Wound Healing: A Mini-Review. GER.

[73] Rønø B., Engelholm L.H., Lund L.R., Hald A. (2013). Gender Affects Skin Wound Healing in Plasminogen Deficient Mice. PLoS ONE.

[74] Pastar I., Stojadinovic O., Yin N.C., Ramirez H., Nusbaum A.G., Sawaya A., Patel S.B., Khalid L., Isseroff R.R., Tomic-Canic M. (2014). Epithelialization in Wound Healing: A Comprehensive Review. Adv. Wound Care.

[75] Hughes O., MacQuhae F., Rakosi A., Herskovitz I., Kirsner R.S., França K., Jafferany M. (2017). Stress and Wound Healing. Stress and Skin Disorders: Basic and Clinical Aspects.

[76] Wynn M., Holloway S. (2019). The Impact of Psychological Stress on Wound Healing: A Theoretical and Clinical Perspective. Wounds UK.

[77] Makrantonaki E., Wlaschek M., Scharffetter-Kochanek K. (2017). Pathogenesis of Wound Healing Disorders in the Elderly. JDDG J. Der Dtsch. Dermatol. Ges..

[78] Pierpont Y.N., Dinh T.P., Salas R.E., Johnson E.L., Wright T.G., Robson M.C., Payne W.G. (2014). Obesity and Surgical Wound Healing: A Current Review. ISRN Obes..

[79] Fairweather M., Heit Y.I., Buie J., Rosenberg L.M., Briggs A., Orgill D.P., Bertagnolli M.M. (2015). Celecoxib Inhibits Early Cutaneous Wound Healing. J. Surg. Res..

[80] Wang A.S., Armstrong E.J., Armstrong A.W. (2013). Corticosteroids and Wound Healing: Clinical Considerations in the Perioperative Period. Am. J. Surg..

[81] Sarkar D., Jung M.K., Wang H.J. (2015). Alcohol and the Immune System. Alcohol. Res..

[82] Lassig A.A.D., Bechtold J.E., Lindgren B.R., Pisansky A., Itabiyi A., Yueh B., Joseph A.M. (2018). Tobacco Exposure and Wound Healing in Head and Neck Surgical Wounds. Laryngoscope.

[83] Larouche J., Sheoran S., Maruyama K., Martino M.M. (2018). Immune Regulation of Skin Wound Healing: Mechanisms and Novel Therapeutic Targets. Adv. Wound Care.

[84] Toczek J., Sadłocha M., Major K., Stojko R. (2022). Benefit of Silver and Gold Nanoparticles in Wound Healing Process after Endometrial Cancer Protocol. Biomedicines.

[85] Leaper D., Assadian O., Edmiston C.E. (2015). Approach to Chronic Wound Infections. Br. J. Dermatol..

[86] Ousey K., Rogers A.A., Rippon M.G. (2016). Hydro-Responsive Wound Dressings Simplify T.I.M.E. Wound Management Framework. Br. J. Community Nurs..

[87] Piemonte G., Benelli L., Braschi F., Rasero L., Matucci-Cerinic M., Denton C.P. (2019). The Local Treatment: Methodology, Debridement and Wound Bed Preparation. Atlas of Ulcers in Systemic Sclerosis: Diagnosis and Management.

[88] Zhang H., Cheng J., Ao Q. (2021). Preparation of Alginate-Based Biomaterials and Their Applications in Biomedicine. Mar. Drugs.

[89] Urtuvia V., Maturana N., Acevedo F., Peña C., Díaz-Barrera A. (2017). Bacterial Alginate Production: An Overview of Its Biosynthesis and Potential Industrial Production. World J. Microbiol. Biotechnol..

[90] Fu S., Thacker A., Sperger D.M., Boni R.L., Buckner I.S., Velankar S., Munson E.J., Block L.H. (2011). Relevance of Rheological Properties of Sodium Alginate in Solution to Calcium Alginate Gel Properties. AAPS PharmSciTech.

[91] Aguero L., Alpdagtas S., Ilhan E., Zaldivar-Silva D., Gunduz O. (2021). Functional Role of Crosslinking in Alginate Scaffold for Drug Delivery and Tissue Engineering: A Review. Eur. Polym. J..

[92] Sanchez-Ballester N.M., Bataille B., Soulairol I. (2021). Sodium Alginate and Alginic Acid as Pharmaceutical Excipients for Tablet Formulation: Structure-Function Relationship. Carbohydr. Polym..

[93] Lim J., Choi G., Joo K.I., Cha H.J., Kim J. (2021). Embolization of Vascular Malformations via In Situ Photocrosslinking of Mechanically Reinforced Alginate Microfibers Using an Optical-Fiber-Integrated Microfluidic Device. Adv. Mater..

[94] Agüero L., Zaldivar-Silva D., Peña L., Dias M.L. (2017). Alginate Microparticles as Oral Colon Drug Delivery Device: A Review. Carbohydr. Polym..

[95] Xu M., Qin M., Cheng Y., Niu X., Kong J., Zhang X., Huang D., Wang H. (2021). Alginate Microgels as Delivery Vehicles for Cell-Based Therapies in Tissue Engineering and Regenerative Medicine. Carbohydr. Polym..

[96] Azarpira N., Kaviani M., Sarvestani F.S. (2021). Incorporation of VEGF-and BFGF-Loaded Alginate Oxide Particles in Acellular Collagen-Alginate Composite Hydrogel to Promote Angiogenesis. Tissue Cell.

[97] Kong X., Chen L., Li B., Quan C., Wu J. (2021). Applications of Oxidized Alginate in Regenerative Medicine. J. Mater. Chem. B.

[98] Cheng L., Yao B., Hu T., Cui X., Shu X., Tang S., Wang R., Wang Y., Liu Y., Song W. (2019). Properties of an Alginate-Gelatin-Based Bioink and Its Potential Impact on Cell Migration, Proliferation, and Differentiation. Int. J. Biol. Macromol..

[99] Mahmoud E.M., Sayed M., El-Kady A.M., Elsayed H., Naga S.M. (2020). In Vitro and in Vivo Study of Naturally Derived Alginate/Hydroxyapatite Bio Composite Scaffolds. Int. J. Biol. Macromol..

[100] Liu W., Madry H., Cucchiarini M. (2022). Application of Alginate Hydrogels for Next-Generation Articular Cartilage Regeneration. Int. J. Mol. Sci..

[101] Grijalvo S., Nieto-Díaz M., Maza R.M., Eritja R., Díaz D.D. (2019). Alginate Hydrogels as Scaffolds and Delivery Systems to Repair the Damaged Spinal Cord. Biotechnol. J..

[102] Wei Q., Zhou J., An Y., Li M., Zhang J., Yang S. (2023). Modification, 3D Printing Process and Application of Sodium Alginate Based Hydrogels in Soft Tissue Engineering: A Review. Int. J. Biol. Macromol..

[103] Xie F., Zou L., Xu Z., Ou X., Guo W., Gao Y., Gao G. (2022). Alginate Foam Gel Modified by Graphene Oxide for Wound Dressing. Int. J. Biol. Macromol..

[104] Vijayan A., Vipin C.L., Kumar G.S.V. (2022). Dual Growth Factor Entrapped Nanoparticle Enriched Alginate Wafer-Based Delivery System for Suppurating Wounds. Int. J. Biol. Macromol..

[105] Najafiasl M., Osfouri S., Azin R., Zaeri S. (2020). Alginate-Based Electrospun Core/Shell Nanofibers Containing Dexpanthenol: A Good Candidate for Wound Dressing. J. Drug. Deliv. Sci. Technol..

[106] Shi M., Zhang H., Song T., Liu X., Gao Y., Zhou J., Li Y. (2019). Sustainable Dual Release of Antibiotic and Growth Factor from PH-Responsive Uniform Alginate Composite Microparticles to Enhance Wound Healing. ACS Appl. Mater. Interfaces.

[107] Choudhary M., Chhabra P., Tyagi A., Singh H. (2021). Scar Free Healing of Full Thickness Diabetic Wounds: A Unique Combination of Silver Nanoparticles as Antimicrobial Agent, Calcium Alginate Nanoparticles as Hemostatic Agent, Fresh Blood as Nutrient/Growth Factor Supplier and Chitosan as Base Matrix. Int. J. Biol. Macromol..

[108] Salehi M., Ehterami A., Farzamfar S., Vaez A., Ebrahimi-Barough S. (2021). Accelerating Healing of Excisional Wound with Alginate Hydrogel Containing Naringenin in Rat Model. Drug. Deliv. Transl. Res..

[109] Aderibigbe B.A., Buyana B. (2018). Alginate in Wound Dressings. Pharmaceutics.

[110] Ehterami A., Salehi M., Farzamfar S., Samadian H., Vaez A., Ghorbani S., Ai J., Sahrapeyma H. (2019). Chitosan/Alginate Hydrogels Containing Alpha-Tocopherol for Wound Healing in Rat Model. J. Drug. Deliv. Sci. Technol..

[111] Dhivya S., Padma V.V., Santhini E. (2015). Wound Dressings—A Review. BioMed.

[112] Varaprasad K., Jayaramudu T., Kanikireddy V., Toro C., Sadiku E.R. (2020). Alginate-Based Composite Materials for Wound Dressing Application:A Mini Review. Carbohydr. Polym..

[113] Sutar T., Bangde P., Dandekar P., Adivarekar R. (2021). Herbal Hemostatic Biopolymeric Dressings of Alginate/Pectin Coated with Croton Oblongifolius Extract. Carbohydr. Polym. Technol. Appl..

[114] Sun X., Ma C., Gong W., Ma Y., Ding Y., Liu L. (2020). Biological Properties of Sulfanilamide-Loaded Alginate Hydrogel Fibers Based on Ionic and Chemical Crosslinking for Wound Dressings. Int. J. Biol. Macromol..

[115] Pelgrift R.Y., Friedman A.J. (2013). Nanotechnology as a Therapeutic Tool to Combat Microbial Resistance. Adv. Drug. Deliv. Rev..

[116] Wahid F., Zhong C., Wang H.-S., Hu X.-H., Chu L.-Q. (2017). Recent Advances in Antimicrobial Hydrogels Containing Metal Ions and Metals/Metal Oxide Nanoparticles. Polymers.

[117] Alavi M., Nokhodchi A. (2020). An Overview on Antimicrobial and Wound Healing Properties of ZnO Nanobiofilms, Hydrogels, and Bionanocomposites Based on Cellulose, Chitosan, and Alginate Polymers. Carbohydr. Polym..

[118] Carpa R., Remizovschi A., Culda C.A., Butiuc-Keul A.L. (2022). Inherent and Composite Hydrogels as Promising Materials to Limit Antimicrobial Resistance. Gels.

[119] Meaume S., Vallet D., Morere M.N., Téot L. (2005). Evaluation of a Silver-Releasing Hydroalginate Dressing in Chronic Wounds with Signs of Local Infection. J. Wound Care.

[120] Woo K.Y., Coutts P.M., Sibbald R.G. (2012). A Randomized Controlled Trial to Evaluate an Antimicrobial Dressing with Silver Alginate Powder for the Management of Chronic Wounds Exhibiting Signs of Critical Colonization. Adv. Ski. Wound Care.

[121] Fong J., Wood F. (2006). Nanocrystalline Silver Dressings in Wound Management: A Review. Int. J. Nanomed..

[122] Seo S.Y., Lee G.H., Lee S.G., Jung S.Y., Lim J.O., Choi J.H. (2012). Alginate-Based Composite Sponge Containing Silver Nanoparticles Synthesized in Situ. Carbohydr. Polym..

[123] Neibert K., Gopishetty V., Grigoryev A., Tokarev I., Al-Hajaj N., Vorstenbosch J., Philip A., Minko S., Maysinger D. (2012). Wound-Healing with Mechanically Robust and Biodegradable Hydrogel Fibers Loaded with Silver Nanoparticles. Adv. Healthc. Mater..

[124] Stojkovska J., Djurdjevic Z., Jancic I., Bufan B., Milenkovic M., Jankovic R., Miskovic-Stankovic V., Obradovic B. (2018). Comparative in Vivo Evaluation of Novel Formulations Based on Alginate and Silver Nanoparticles for Wound Treatments. J. Biomater. Appl..

[125] Cai J., Chen X., Wang X., Tan Y., Ye D., Jia Y., Liu P., Yu H. (2018). High-Water-Absorbing Calcium Alginate Fibrous Scaffold Fabricated by Microfluidic Spinning for Use in Chronic Wound Dressings. RSC Adv..

[126] Hu W.-W., Lin Y.-T. (2022). Alginate/Polycaprolactone Composite Fibers as Multifunctional Wound Dressings. Carbohydr. Polym..

[127] Mokhena T.C., Luyt A.S. (2017). Electrospun Alginate Nanofibres Impregnated with Silver Nanoparticles: Preparation, Morphology and Antibacterial Properties. Carbohydr. Polym..

[128] Huang L., Yu L., Yin X., Lin Y., Xu Y., Niu Y. (2022). Silver Nanoparticles with Vanadium Oxide Nanowires Loaded into Electrospun Dressings for Efficient Healing of Bacterium-Infected Wounds. J. Colloid Interface Sci..

[129] Linhart A.N., Wortman-Otto K.M., Deninger I., Dudek A.L., Lange H.R., Danhausen D.M., Graverson C.F., Beckmann T.J., Havens M.A., Keleher J.J. (2020). Strategic Design of Antimicrobial Hydrogels Containing Biomimetic Additives for Enhanced Matrix Responsiveness and HDFa Wound Healing Rates. ACS Appl. Bio Mater..

[130] Ambrogi V., Pietrella D., Donnadio A., Latterini L., Di Michele A., Luffarelli I., Ricci M. (2020). Biocompatible Alginate Silica Supported Silver Nanoparticles Composite Films for Wound Dressing with Antibiofilm Activity. Mater. Sci. Eng. C.

[131] Shin J.U., Gwon J., Lee S.-Y., Yoo H.S. (2018). Silver-Incorporated Nanocellulose Fibers for Antibacterial Hydrogels. ACS Omega.

[132] Liang L., Hou T., Ouyang Q., Xie L., Zhong S., Li P., Li S., Li C. (2020). Antimicrobial Sodium Alginate Dressing Immobilized with Polydopamine-Silver Composite Nanospheres. Compos. Part B Eng..

[133] Zhou M., Lin F., Li W., Shi L., Li Y., Shan G. (2021). Development of Nanosilver Doped Carboxymethyl Chitosan-Polyamideamine Alginate Composite Dressing for Wound Treatment. Int. J. Biol. Macromol..

[134] Gómez Chabala L.F., Cuartas C.E.E., López M.E.L. (2017). Release Behavior and Antibacterial Activity of Chitosan/Alginate Blends with Aloe Vera and Silver Nanoparticles. Mar. Drugs.

[135] Gordienko M.G., Palchikova V.V., Kalenov S.V., Lebedev E.A., Belov A.A., Menshutina N.V. (2022). The Alginate–Chitosan Composite Sponges with Biogenic Ag Nanoparticles Produced by Combining of Cryostructuration, Ionotropic Gelation and Ion Replacement Methods. Int. J. Polym. Mater. Polym. Biomater..

[136] Chalitangkoon J., Wongkittisin M., Monvisade P. (2020). Silver Loaded Hydroxyethylacryl Chitosan/Sodium Alginate Hydrogel Films for Controlled Drug Release Wound Dressings. Int. J. Biol. Macromol..

[137] Ma L., Tan Y., Chen X., Ran Y., Tong Q., Tang L., Su W., Wang X., Li X. (2022). Injectable Oxidized Alginate/Carboxylmethyl Chitosan Hydrogels Functionalized with Nanoparticles for Wound Repair. Carbohydr. Polym..

[138] Abou-Okeil A., Fahmy H.M., El-Bisi M.K., Ahmed-Farid O.A. (2018). Hyaluronic Acid/Na-Alginate Films as Topical Bioactive Wound Dressings. Eur. Polym. J..

[139] Tarusha L., Paoletti S., Travan A., Marsich E. (2018). Alginate Membranes Loaded with Hyaluronic Acid and Silver Nanoparticles to Foster Tissue Healing and to Control Bacterial Contamination of Non-Healing Wounds. J. Mater. Sci. Mater. Med..

[140] Catanzano O., D’Esposito V., Pulcrano G., Maiolino S., Ambrosio M.R., Esposito M., Miro A., Ungaro F., Formisano P., Catania M.R. (2017). Ultrasmall Silver Nanoparticles Loaded in Alginate–Hyaluronic Acid Hybrid Hydrogels for Treating Infected Wounds. Int. J. Polym. Mater. Polym. Biomater..

[141] Pankongadisak P., Ruktanonchai U.R., Supaphol P., Suwantong O. (2014). Preparation and Characterization of Silver Nanoparticles-Loaded Calcium Alginate Beads Embedded in Gelatin Scaffolds. AAPS PharmSciTech.

[142] Baukum J., Pranjan J., Kaolaor A., Chuysinuan P., Suwantong O., Supaphol P. (2020). The Potential Use of Cross-Linked Alginate/Gelatin Hydrogels Containing Silver Nanoparticles for Wound Dressing Applications. Polym. Bull..

[143] Diniz F.R., Maia R.C.A.P., de Andrade L.R.M., Andrade L.N., Vinicius Chaud M., da Silva C.F., Corrêa C.B., de Albuquerque Junior R.L.C., Pereira da Costa L., Shin S.R. (2020). Silver Nanoparticles-Composing Alginate/Gelatine Hydrogel Improves Wound Healing In Vivo. Nanomaterials.

[144] Zhang H., Peng M., Cheng T., Zhao P., Qiu L., Zhou J., Lu G., Chen J. (2018). Silver Nanoparticles-Doped Collagen–Alginate Antimicrobial Biocomposite as Potential Wound Dressing. J. Mater. Sci..

[145] Tao G., Cai R., Wang Y., Zuo H., He H. (2021). Fabrication of Antibacterial Sericin Based Hydrogel as an Injectable and Mouldable Wound Dressing. Mater. Sci. Eng. C.

[146] Vidovic S., Stojkovska J., Stevanovic M., Balanc B., Vukasinovic-Sekulic M., Marinkovic A., Obradovic B. (2022). Effects of Poly(Vinyl Alcohol) Blending with Ag/Alginate Solutions to Form Nanocomposite Fibres for Potential Use as Antibacterial Wound Dressings. R. Soc. Open Sci..

[147] Chen K., Wang F., Liu S., Wu X., Xu L., Zhang D. (2020). In Situ Reduction of Silver Nanoparticles by Sodium Alginate to Obtain Silver-Loaded Composite Wound Dressing with Enhanced Mechanical and Antimicrobial Property. Int. J. Biol. Macromol..

[148] Kong F., Fan C., Yang Y., Lee B.H., Wei K. (2019). 5-Hydroxymethylfurfural-Embedded Poly (Vinyl Alcohol)/Sodium Alginate Hybrid Hydrogels Accelerate Wound Healing. Int. J. Biol. Macromol..

[149] Singh R., Singh D. (2012). Radiation Synthesis of PVP/Alginate Hydrogel Containing Nanosilver as Wound Dressing. J. Mater. Sci. Mater. Med..

[150] Król A., Pomastowski P., Rafińska K., Railean-Plugaru V., Buszewski B. (2017). Zinc Oxide Nanoparticles: Synthesis, Antiseptic Activity and Toxicity Mechanism. Adv. Colloid. Interface Sci..

[151] Loera-Valencia R., Neira R.E., Urbina B.P., Camacho A., Galindo R.B. (2022). Evaluation of the Therapeutic Efficacy of Dressings with ZnO Nanoparticles in the Treatment of Diabetic Foot Ulcers. Biomed. Pharm..

[152] Cleetus C.M., Alvarez Primo F., Fregoso G., Lalitha Raveendran N., Noveron J.C., Spencer C.T., Ramana C.V., Joddar B. (2020). Alginate Hydrogels with Embedded ZnO Nanoparticles for Wound Healing Therapy. Int. J. Nanomed..

[153] Rahman M.A., Islam M.S., Haque P., Khan M.N., Takafuji M., Begum M., Chowdhury G.W., Khan M., Rahman M.M. (2020). Calcium Ion Mediated Rapid Wound Healing by Nano-ZnO Doped Calcium Phosphate-Chitosan-Alginate Biocomposites. Materialia.

[154] Nozari M., Gholizadeh M., Zahiri Oghani F., Tahvildari K. (2021). Studies on Novel Chitosan/Alginate and Chitosan/Bentonite Flexible Films Incorporated with ZnO Nano Particles for Accelerating Dermal Burn Healing: In Vivo and in Vitro Evaluation. Int. J. Biol. Macromol..

[155] Mohandas A., Pt S.K., Raja B., Lakshmanan V.-K., Jayakumar R. (2015). Exploration of Alginate Hydrogel/Nano Zinc Oxide Composite Bandages for Infected Wounds. Int. J. Nanomed..

[156] Zhang M., Qiao X., Han W., Jiang T., Liu F., Zhao X. (2021). Alginate-Chitosan Oligosaccharide-ZnO Composite Hydrogel for Accelerating Wound Healing. Carbohydr. Polym..

[157] Arshad R., Sohail M.F., Sarwar H.S., Saeed H., Ali I., Akhtar S., Hussain S.Z., Afzal I., Jahan S., Anees-ur-Rehman (2019). ZnO-NPs Embedded Biodegradable Thiolated Bandage for Postoperative Surgical Site Infection: In Vitro and in Vivo Evaluation. PLoS ONE.

[158] Zhu J., Jiang G., Song G., Liu T., Cao C., Yang Y., Zhang Y., Hong W. (2019). Incorporation of ZnO/Bioactive Glass Nanoparticles into Alginate/Chitosan Composite Hydrogels for Wound Closure. ACS Appl. Bio Mater..

[159] Raguvaran R., Manuja B.K., Chopra M., Thakur R., Anand T., Kalia A., Manuja A. (2017). Sodium Alginate and Gum Acacia Hydrogels of ZnO Nanoparticles Show Wound Healing Effect on Fibroblast Cells. Int. J. Biol. Macromol..

[160] Manuja A., Raguvaran R., Kumar B., Kalia A., Tripathi B.N. (2020). Accelerated Healing of Full Thickness Excised Skin Wound in Rabbits Using Single Application of Alginate/Acacia Based Nanocomposites of ZnO Nanoparticles. Int. J. Biol. Macromol..

[161] Buyana B., Aderibigbe B.A., Ndinteh D.T., Fonkui Y.T., Kumar P. (2020). Alginate-Pluronic Topical Gels Loaded with Thymol, Norfloxacin and ZnO Nanoparticles as Potential Wound Dressings. J. Drug Deliv. Sci. Technol..

[162] Ndlovu S.P., Fonkui T.Y., Ndinteh D.T., Aderibigbe B.A. (2022). Dissolvable Wound Dressing Loaded with Silver Nanoparticles Together with Ampicillin and Ciprofloxacin. Ther. Deliv..

[163] Shalumon K.T., Anulekha K.H., Nair S.V., Nair S.V., Chennazhi K.P., Jayakumar R. (2011). Sodium Alginate/Poly(Vinyl Alcohol)/Nano ZnO Composite Nanofibers for Antibacterial Wound Dressings. Int. J. Biol. Macromol..

[164] Dodero A., Scarfi S., Pozzolini M., Vicini S., Alloisio M., Castellano M. (2020). Alginate-Based Electrospun Membranes Containing ZnO Nanoparticles as Potential Wound Healing Patches: Biological, Mechanical, and Physicochemical Characterization. ACS Appl. Mater. Interfaces.

[165] Dodero A., Alloisio M., Castellano M., Vicini S. (2020). Multilayer Alginate–Polycaprolactone Electrospun Membranes as Skin Wound Patches with Drug Delivery Abilities. ACS Appl. Mater. Interfaces.

[166] Gutierrez E., Burdiles P.A., Quero F., Palma P., Olate-Moya F., Palza H. (2019). 3D Printing of Antimicrobial Alginate/Bacterial-Cellulose Composite Hydrogels by Incorporating Copper Nanostructures. ACS Biomater. Sci. Eng..

[167] Maheswary T., Nurul A.A., Fauzi M.B. (2021). The Insights of Microbes’ Roles in Wound Healing: A Comprehensive Review. Pharmaceutics.

[168] Punjataewakupt A., Napavichayanun S., Aramwit P. (2019). The Downside of Antimicrobial Agents for Wound Healing. Eur. J. Clin. Microbiol. Infect. Dis..

[169] Alzarea A.I., Alruwaili N.K., Ahmad M.M., Munir M.U., Butt A.M., Alrowaili Z.A., Shahari M.S.B., Almalki Z.S., Alqahtani S.S., Dolzhenko A.V. (2022). Development and Characterization of Gentamicin-Loaded Arabinoxylan-Sodium Alginate Films as Antibacterial Wound Dressing. Int. J. Mol. Sci..

[170] Shahzad A., Khan A., Afzal Z., Umer M.F., Khan J., Khan G.M. (2019). Formulation Development and Characterization of Cefazolin Nanoparticles-Loaded Cross-Linked Films of Sodium Alginate and Pectin as Wound Dressings. Int. J. Biol. Macromol..

[171] Reczyńska-Kolman K., Hartman K., Kwiecień K., Brzychczy-Włoch M., Pamuła E. (2022). Composites Based on Gellan Gum, Alginate and Nisin-Enriched Lipid Nanoparticles for the Treatment of Infected Wounds. Int. J. Mol. Sci..

[172] Fan Y., Wu W., Lei Y., Gaucher C., Pei S., Zhang J., Xia X. (2019). Edaravone-Loaded Alginate-Based Nanocomposite Hydrogel Accelerated Chronic Wound Healing in Diabetic Mice. Mar. Drugs.

[173] Atia N.M., Hazzah H.A., Gaafar P.M.E., Abdallah O.Y. (2019). Diosmin Nanocrystal–Loaded Wafers for Treatment of Diabetic Ulcer: In Vitro and In Vivo Evaluation. J. Pharm. Sci..

[174] Monou P.K., Mamaligka A.M., Tzimtzimis E.K., Tzetzis D., Vergkizi-Nikolakaki S., Vizirianakis I.S., Andriotis E.G., Eleftheriadis G.K., Fatouros D.G. (2022). Fabrication and Preliminary In Vitro Evaluation of 3D-Printed Alginate Films with Cannabidiol (CBD) and Cannabigerol (CBG) Nanoparticles for Potential Wound-Healing Applications. Pharmaceutics.

[175] Guadarrama-Acevedo M.C., Mendoza-Flores R.A., Del Prado-Audelo M.L., Urbán-Morlán Z., Giraldo-Gomez D.M., Magaña J.J., González-Torres M., Reyes-Hernández O.D., Figueroa-González G., Caballero-Florán I.H. (2019). Development and Evaluation of Alginate Membranes with Curcumin-Loaded Nanoparticles for Potential Wound-Healing Applications. Pharmaceutics.

[176] Saygili E., Kaya E., Ilhan-Ayisigi E., Saglam-Metiner P., Alarcin E., Kazan A., Girgic E., Kim Y.-W., Gunes K., Eren-Ozcan G.G. (2021). An Alginate-Poly(Acrylamide) Hydrogel with TGF-Β3 Loaded Nanoparticles for Cartilage Repair: Biodegradability, Biocompatibility and Protein Adsorption. Int. J. Biol. Macromol..

[177] Lin X., Guan X., Wu Y., Zhuang S., Wu Y., Du L., Zhao J., Rong J., Zhao J., Tu M. (2020). An Alginate/Poly(N-Isopropylacrylamide)-Based Composite Hydrogel Dressing with Stepwise Delivery of Drug and Growth Factor for Wound Repair. Mater. Sci. Eng. C.

[178] Mihai M.M., Dima M.B., Dima B., Holban A.M. (2019). Nanomaterials for Wound Healing and Infection Control. Materials.

[179] Pacho M.N., Manzano V.E., D’Accorso N.B., Hasnain M.S., Nayak A.K. (2019). Chapter 16—Synthesis of Micro- and Nanoparticles of Alginate and Chitosan for Controlled Release of Drugs. Natural Polysaccharides in Drug Delivery and Biomedical Applications.

[180] Del Gaudio P., Amante C., Civale R., Bizzarro V., Petrella A., Pepe G., Campiglia P., Russo P., Aquino R.P. (2020). In Situ Gelling Alginate-Pectin Blend Particles Loaded with Ac2-26: A New Weapon to Improve Wound Care Armamentarium. Carbohydr. Polym..

[181] Oliveira D.M.L., Rezende P.S., Barbosa T.C., Andrade L.N., Bani C., Tavares D.S., da Silva C.F., Chaud M.V., Padilha F., Cano A. (2020). Double Membrane Based on Lidocaine-Coated Polymyxin-Alginate Nanoparticles for Wound Healing: In Vitro Characterization and in Vivo Tissue Repair. Int. J. Pharm..

[182] Wu X., Zhang Q., Wang Z., Xu Y., Tao Q., Wang J., Kong X., Sheng K., Wang Y. (2022). Investigation of Construction and Characterization of Carboxymethyl Chitosan—Sodium Alginate Nanoparticles to Stabilize Pickering Emulsion Hydrogels for Curcumin Encapsulation and Accelerating Wound Healing. Int. J. Biol. Macromol..

[183] Sheir M.M., Nasra M.M.A., Abdallah O.Y. (2021). Chitosan Alginate Nanoparticles as a Platform for the Treatment of Diabetic and Non-Diabetic Pressure Ulcers: Formulation and in Vitro/in Vivo Evaluation. Int. J. Pharm..

[184] Montaser A.S., Abdel-Mohsen A.M., Ramadan M.A., Sleem A.A., Sahffie N.M., Jancar J., Hebeish A. (2016). Preparation and Characterization of Alginate/Silver/Nicotinamide Nanocomposites for Treating Diabetic Wounds. Int. J. Biol. Macromol..

[185] Maatouk B., Jaffa M.A., Karam M., Fahs D., Nour-Eldine W., Hasan A., Jaffa A.A., Mhanna R. (2021). Sulfated Alginate/Polycaprolactone Double-Emulsion Nanoparticles for Enhanced Delivery of Heparin-Binding Growth Factors in Wound Healing Applications. Colloids Surf. B Biointerfaces.

[186] Hosny A.E.-D.M., Rasmy S.A., Aboul-Magd D.S., Kashef M.T., El-Bazza Z.E. (2019). The Increasing Threat of Silver-Resistance in Clinical Isolates from Wounds and Burns. Infect. Drug. Resist..

[187] Percival S.L., Salisbury A.-M., Chen R. (2019). Silver, Biofilms and Wounds: Resistance Revisited. Crit. Rev. Microbiol..

[188] Catanzano O., Quaglia F., Boateng J.S. (2021). Wound Dressings as Growth Factor Delivery Platforms for Chronic Wound Healing. Expert. Opin. Drug. Deliv..

[189] Bierhalz A.C.K., Moraes Â.M. (2017). Composite Membranes of Alginate and Chitosan Reinforced with Cotton or Linen Fibers Incorporating Epidermal Growth Factor. Mater. Sci. Eng. C.

[190] Zeng H.-Y., Huang Y.-C. (2018). Basic Fibroblast Growth Factor Released from Fucoidan-Modified Chitosan/Alginate Scaffolds for Promoting Fibroblasts Migration. J. Polym. Res..

[191] Mohammadi S., Ramakrishna S., Laurent S., Shokrgozar M.A., Semnani D., Sadeghi D., Bonakdar S., Akbari M. (2019). Fabrication of Nanofibrous PVA/Alginate-Sulfate Substrates for Growth Factor Delivery. J. Biomed. Mater. Res. Part A.

[192] McCarthy S., Dvorakova V., O’Sullivan P., Bourke J.F. (2018). Anaphylaxis Caused by Alginate Dressing. Contact Dermat..

[193] Ionita M., Pandele M.A., Iovu H. (2013). Sodium Alginate/Graphene Oxide Composite Films with Enhanced Thermal and Mechanical Properties. Carbohydr. Polym..

[194] Song J.-L., Fu X.-Y., Raza A., Shen N.-A., Xue Y.-Q., Wang H.-J., Wang J.-Y. (2020). Enhancement of Mechanical Strength of TCP-Alginate Based Bioprinted Constructs. J. Mech. Behav. Biomed. Mater..

[195] Stoppel W.L., White J.C., Horava S.D., Henry A.C., Roberts S.C., Bhatia S.R. (2014). Terminal Sterilization of Alginate Hydrogels: Efficacy and Impact on Mechanical Properties. J. Biomed. Mater. Res. Part B Appl. Biomater..

[196] Fu P.-S., Wang J.-C., Lai P.-L., Liu S.-M., Chen Y.-S., Chen W.-C., Hung C.-C. (2021). Effects of Gamma Radiation on the Sterility Assurance, Antibacterial Ability, and Biocompatibility of Impregnated Hydrogel Macrosphere Protein and Drug Release. Polymers.

[197] Mollah M.Z.I., Rahaman M.S., Faruque M.R.I., Khandaker M.U., Osman H., Alamri S., Al-Assaf S. (2021). Effects of Radiation Sterilization Dose on the Molecular Weight and Gelling Properties of Commercial Alginate Samples. Front. Mater..

[198] Bernhardt A., Wehrl M., Paul B., Hochmuth T., Schumacher M., Schütz K., Gelinsky M. (2015). Improved Sterilization of Sensitive Biomaterials with Supercritical Carbon Dioxide at Low Temperature. PLoS ONE.

